# Dual Targeting of Alcohol Use Disorder and Alcohol–Associated Liver Disease: Emerging Molecular Targets Along the Gut–Liver–Brain Axis

**DOI:** 10.3390/ijms27219921

**Published:** 2026-11-05

**Authors:** Tom Ryu, Beom Sun Chung, Keungmo Yang

**Affiliations:** 1 Department of Internal Medicine, Institute for Digestive Research, Digestive Disease Center, Soonchunhyang University College of Medicine, Seoul 04401, Republic of Korea; tomryu1@schmc.ac.kr; 2 Department of Anatomy, Yonsei University Wonju College of Medicine, Wonju 26426, Republic of Korea; bschung@yonsei.ac.kr; 3 Department of Internal Medicine, Division of Gastroenterology and Hepatology, College of Medicine, The Catholic University of Korea, Seoul 06591, Republic of Korea

**Keywords:** alcohol–associated liver disease, alcohol use disorder, gut–liver–brain axis, GLP-1 receptor, FGF21, mGluR5, gut microbiota, molecular targets

## Abstract

Alcohol–associated liver disease (ALD) is the hepatic end of alcohol use disorder (AUD), yet only abstinence and transplantation modify it, and recent liver-only programmes in alcohol–associated hepatitis have not delivered. This review asks whether the receptors, ligands and transporters setting drinking behaviour and alcohol-related liver injury are largely one set along the gut–liver–brain axis, so one molecule could lower both. Where resolved, it is cell-specific: hepatic glucagon-like peptide-1 receptor sits, in non-alcohol models, on sinusoidal endothelial and CD8^+^ T cells, not hepatocytes, and ethanol induces hepatocyte fibroblast growth factor 21, whose analogue acts on β-Klotho^+^ amygdala neurons. Organised by target, not organ, each candidate is appraised separately on both sides, negatives, terminations and non-replications counting alike. Of sixteen targets spanning incretin, hepatokine, glutamatergic and microbial or neuroimmune families, three are supported on both sides in the desired direction but only in separate experiments, three point in opposite directions, and ten have had no manipulation read out on both; for none were both organs read out in one experiment. Dual targeting changes what trials must measure: only one of 64 registered trials makes both endpoints primary, so objectively measured intake and a liver endpoint must be co-primary.

## 1. Introduction

Alcohol–associated liver disease (ALD) is the hepatic end of a far commoner condition. The Global Burden of Disease 2021 analysis counted 111.12 million cases of alcohol use disorder (AUD) against 3.02 million of ALD, and between 2019 and 2021 ALD prevalence, incidence and mortality rose more in women than in men [1]. The behavioural disorder is the larger reservoir, and least treated where hepatic risk is greatest: in commercially insured US adults with AUD, pharmacotherapy reached 14.5% without ALD but 2.3% with ALD cirrhosis [2].

Short of transplantation, abstinence remains the only intervention that alters the liver disease itself. Guidelines name it the crucial determinant of long-term outcome, and leave corticosteroids the only agent of proven short-term survival benefit in severe alcohol–associated hepatitis (AH) [3]. In 344 patients with decompensated cirrhosis of steatotic liver disease, not alcohol-related cirrhosis alone, continued drinking carried a 5.3-fold mortality hazard, and abstinence corroborated by the blood biomarker phosphatidylethanol (PEth) independently predicted recompensation [4]. Recompensation was reached by only 18.6%, and Baveno VII defines it to include removal of the primary aetiological factor, so that estimate quantifies the association rather than isolating abstinence [4]. Treating the behaviour tracks the organ: in 17,548 propensity-matched patients with alcohol-related cirrhosis and persistent drinking, AUD medication was associated with lower three-year mortality (21.6% vs. 29.3%)—a health-record association, not a trial result [5].

Nomenclature followed. The 2023 multi-society Delphi statement made steatotic liver disease the umbrella term, renamed its metabolic form metabolic dysfunction–associated steatotic liver disease (MASLD), and created metabolic dysfunction–associated alcohol-related liver disease (MetALD) for people meeting a metabolic criterion who drink 140–350 g (women) or 210–420 g (men) weekly [6]. Those thresholds rest on self-report: PEth was positive in 69.4% of patients labelled MetALD and negative in those labelled MASLD [4]. The criteria are contested on a sharper ground, that heavy drinking itself produces the cardiometabolic criteria used to diagnose MASLD, so one criterion above those thresholds may misclassify alcohol-driven disease as metabolic [7]. MetALD names the population a dual-acting molecule would reach first.

Drug development has nevertheless proceeded one organ at a time, and recent liver-only programmes in AH have not delivered. Larsucosterol missed its 90-day death-or-transplantation endpoint in a phase 2b trial of 307 patients [8]. Anakinra with zinc was stopped early because prednisone gave higher 90-day survival, 90% vs. 70% [9]. Canakinumab showed no significant benefit on paired biopsies [10]. These are examples, not a census; none measured drinking. The premise examined here is that the receptors, ligands and transporters setting drinking behaviour and those setting alcohol-related liver injury are largely one set along the gut–liver–brain axis, testable on both sides. That set is now resolved, in places, to named molecules on identified cells. The hepatic glucagon-like peptide-1 receptor (GLP-1R) sits on pericentral liver sinusoidal endothelial cells and CD8^+^ T cells rather than hepatocytes [11], while a dorsal-septal GLP-1R population carries the drinking effect [12]. Hepatokines cross the other way: ethanol induces hepatocyte fibroblast growth factor 21 (FGF21) through its own catabolites [13], and the hormone acts on β-Klotho-expressing basolateral amygdala neurons to lower intake [14]. Glutamatergic signalling supplies a third route, hepatocyte vesicular glutamate transporter 3 (VGLUT3)-dependent release acting on Kupffer-cell metabotropic glutamate receptor 5 (mGluR5) and NADPH oxidase 2 (NOX2) [15]. The gut supplies a fourth: a microbiota-derived aryl-hydrocarbon-receptor ligand, indole-3-acetic acid, sets innate-lymphoid-cell interleukin (IL)-22 and intestinal REG3G [16]. And the wiring is anatomical as well as humoral—hepatic vagal afferents report the intestinal microenvironment to the nucleus tractus solitarius [17], and the anti-drinking effect of a peripherally restricted cannabinoid receptor type 1 (CB1) antagonist requires CB1 on Gpr65^+^ nodose-ganglion afferents rather than brain neurons [18].

Several reviews come close. Wolstenholme et al. frame the same axis in AUD but test one modality, faecal microbiota transplantation, with no receptor, hepatokine or transporter target and a 2022 search [19]. The dual-target principle is credited, not claimed: Kharbanda et al. stated it plainly for the ghrelin system, before the 2023–2026 incretin, hepatokine and genetic data [20]. A redox-centred reading extended to MetALD, by two of the present authors, already proposes a glutamate-based liver–brain “metabolic synapse” [21]; that proposal is treated here as prior work, and Section 5 claims only the cell pair, the release mechanism and the structural requirement. The in-journal comparator covers GLP-1 receptor agonists in AUD and does contain a dedicated ALD section, built on two rodent NIAAA-model studies and database analyses, but organised as central pharmacology for one drug class [22]. Several further reviews read the same axis through single mediators or single modalities—among them the molecular basis of abstinence and relapse in ALD [23] and bile-acid signalling from liver to drinking behaviour [24]—none of them appraising target by target. To the in-journal comparator this review adds, beyond the mechanisms above, genetics separating *GLP1R* from *GIPR*, the randomised series with its negative and failed-primary results, separability from weight loss, three further target families, an appraisal that ranks pharmaco-epidemiology below randomised trials, and the dual-endpoint design argument.

**Figure 1:**
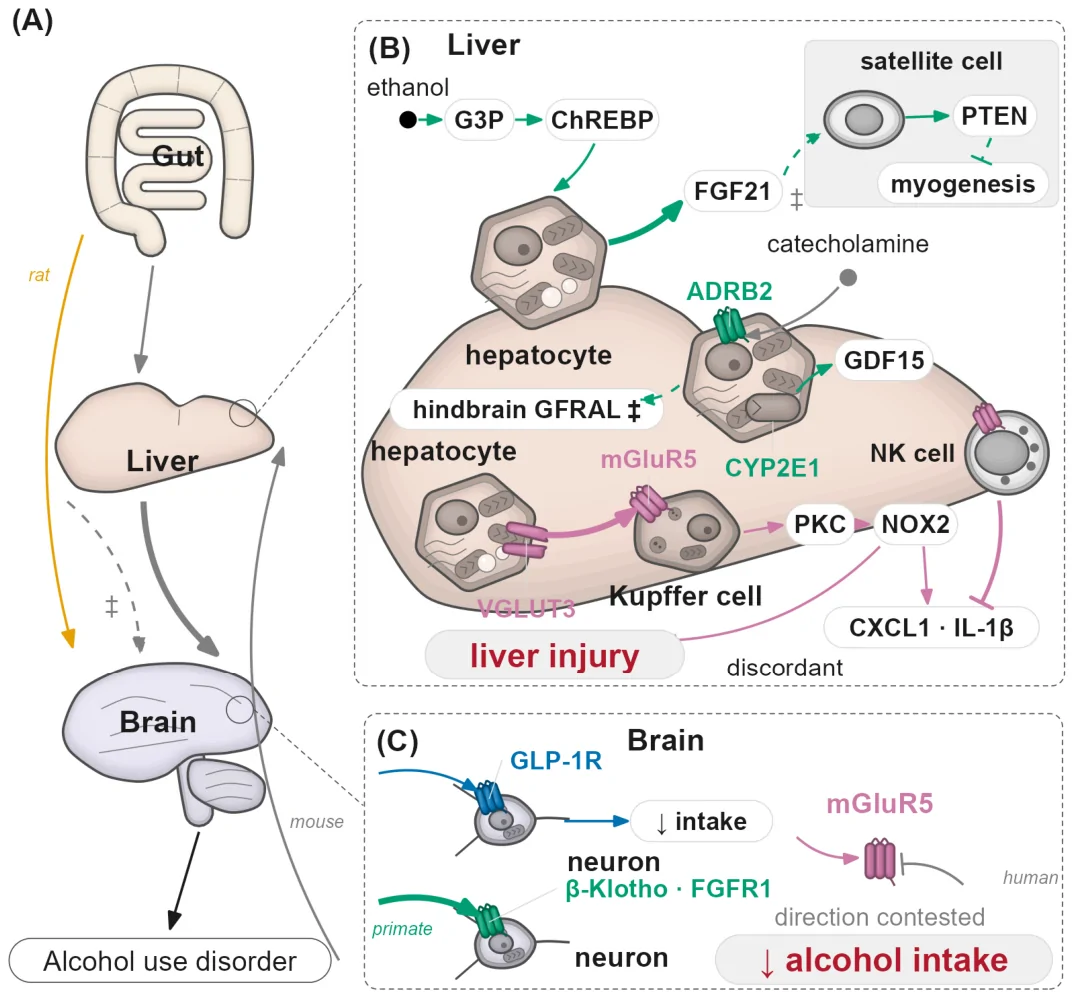
Three molecular families act at defined cells on both the hepatic and the behavioural side of the gut–liver–brain axis. (**A**) The axis, read from gut at the top to brain at the bottom, and its routes: ethanol-associated barrier permeability delivers luminal contents to the portal circulation, a vagus-dependent microbial route carries signals to the brain, hepatokines travel humorally, a central node sets voluntary intake [12], and early alcohol-induced steatosis preceded higher subsequent intake, correlated with its degree (female mice) [25], so the loop runs both ways. (**B**) The liver callout, drawn on the liver body as a compartment holding its cells: ethanol induces hepatocyte FGF21 through G3P and ChREBP, and the dashed ‡ arm to muscle satellite cells, where PTEN blocks myogenesis, marks the discordant muscle arm; gut-derived catecholamine, which belongs to no target family and is drawn in the neutral grey register, acts at hepatocyte ADRB2 and mitochondrial CYP2E1 to raise GDF15, which drives Kupffer-cell death and endotoxin tolerance and reaches hindbrain GFRAL (dashed, ‡); and hepatocyte VGLUT3 releases glutamate onto Kupffer-cell mGluR5, signalling through PKC and NOX2 and branching to CXCL1 · IL-1β and to liver injury, while the same receptor on natural-killer cells blocks that branch—the opposite sign of one receptor. (**C**) The brain callout: GLP-1R on a neuron lowers intake, FGF21 acts at β-Klotho · FGFR1 neurons (primate), and human mGluR5 availability is drawn as two opposed arrows because its direction is contested. Solid arrows denote signalling shown in an alcohol model or in humans; flat heads denote inhibition; dashed arrows marked ‡ rest only on a non-alcohol model or endpoint; two opposed arrows meeting at one node are labelled “discordant” or “direction contested”. Italic *rat*, *mouse*, *primate* and *human* mark the four places where the species matters to the mechanism. *Abbreviations:* ADRB2, β2-adrenergic receptor; ChREBP, carbohydrate-response-element-binding protein; CXCL1, C-X-C motif chemokine ligand 1; CYP2E1, cytochrome P450 2E1; FGFR1, fibroblast growth factor receptor 1; G3P, glycerol-3-phosphate; GDF15, growth differentiation factor 15; GFRAL, glial cell line-derived neurotrophic factor (GDNF)-family receptor α-like; IL-1β, interleukin-1β; NK, natural killer; PKC, protein kinase C; PTEN, phosphatase and tensin homolog. The evidence, species, model and citation for every element of (**B**,**C**) are given in Table S1 and in Section 3, Section 4, Section 5 and Section 6.

What is new is a method rather than a molecule: each candidate is appraised separately on the drinking side and on the liver side, negative and non-replicated results weighed alongside the positive ones, placed on its cells of origin and action (Figure 1) and assembled in one evidence matrix (Table S1). Section 2 gives the routes of the axis and what it takes for a target to count as dual; Section 3, Section 4, Section 5 and Section 6 apply that reading to the four target families; Section 7 and Section 8 read across the targets and name the decisive experiments.

Several areas are excluded by design, so their absence is not an omission. Three classes of evidence are out of scope: evidence confined to MASLD or metabolic dysfunction–associated steatohepatitis (MASH) unless labelled a liver-side benchmark; alcohol-metabolising enzymology, oxidative stress and the inflammasome as organising themes; and clinical management, approved AUD pharmacotherapy as practice and transplantation policy. So are hepatic encephalopathy and alcohol-related brain injury as entities, the brain here being a control node for drinking rather than an injured organ. Excluded as topics are carcinogenesis, nutritional preparations, device interventions, paediatric exposure and alcohol policy.

## 2. The Gut–Liver–Brain Axis and What Counts as a Dual Target

### 2.1. Routes of Communication Along the Axis

Communication along the gut–liver–brain axis runs through two routes: humoral signalling by metabolites, hepatokines, toxins and inflammatory mediators, and neural signalling by vagal afferents in the common hepatic branch—the arm more likely to explain the behavioural consequences of chronic liver disease (Figure 1) [26].

In mice, hepatic vagal sensory afferents report the intestinal microenvironment to the nucleus tractus solitarius, and the same reflex’s efferent limb sustains colonic regulatory T cells through muscarinic induction of aldehyde dehydrogenase and retinoic acid, so cutting the afferents contracts that pool [17]. The same route can set a consummatory behaviour: hepatocyte clock disruption in mice altered food-intake patterning, corrected by ablating the hepatic vagal afferent nerve—liver-to-brain control of ingestion, measured for food, not alcohol [27]. One experiment joins both routes to a drinking endpoint: bacterial extracellular vesicles from the microbiota of high-drinking rats raised voluntary alcohol intake up to ten-fold in alcohol-rejecting recipients, without systemic or brain inflammation, and vagotomy abolished the transfer [28].

The axis’s barriers are permissive rather than instructive: they set how much signal crosses, not what it says. Gut permeability was raised in only a subset of alcohol-dependent patients, who carried an altered microbiota and craving; anxiety and depression scores persisting after three weeks of abstinence [29]. At the far end of the axis, faeces from patients with AUD transferred into germ-free mice increased cerebral tracer leakage and lowered ZO-1, occludin and claudin-5 [30]. Routes locate where a cross-organ drug could act; only the molecule at either end can be appraised as a target. A route can carry a signal without an inflammatory intermediate; Section 6.3’s immune accounts are one mechanism, not a requirement.

### 2.2. What Counts as a Dual Target

A target counts as dual here only if there is evidence on both the behavioural/central and the hepatic side, and each side is appraised on its own, since a strong literature on one organ says nothing about the other. The two sides are therefore described separately for every candidate in Section 3, Section 4, Section 5 and Section 6 and set beside each other in the evidence matrix (Table S1), where negative, terminated and non-replicated results are given the same prominence as positive ones.

What such evidence can show differs in kind as much as in strength. Expression, correlation and association data, in humans or in animals and with no manipulation of the target, locate a molecule without implicating it. A loss- or gain-of-function manipulation—knockout, conditional knockout, agonist, antagonist, small interfering RNA—that changes the organ-relevant phenotype in a defined model, with the cell type identified, implicates it in that model. Human genetic evidence (association, rare variant, Mendelian randomisation), a validated human biomarker response, and prospective-equivalent observational designs, such as pharmaco-epidemiology and target-trial emulation, carry a claim into people, but none of them is an experiment, and none is read here as a substitute for a trial. The most that can be shown is a prospective human trial in which engaging the target pharmacologically changes a clinical drinking outcome over a treatment period—heavy drinking days, drinks per week, abstinence or PEth—or a liver outcome, meaning histology, decompensation or survival. A serum or imaging fibrosis marker is a biomarker endpoint; however, a trial designates it, and single-session laboratory, cue-reactivity and craving readouts show target engagement rather than a clinical outcome, the paradigm’s predictive validity for medication development being itself under appraisal [31].

Failure is read as carefully as success. A negative trial of adequate size caps what the clinical literature can show for that agent without undoing the mechanism beneath it. A stopped programme records a decision, with the registry reason, rather than a result. An interventional study that could not inform—terminated, aborted or underpowered—establishes nothing either way and is reported with the reason it could not inform; where a replication fails, the human claim is treated as associative again and the failure is stated beside the original. Several further cautions run through the sections that follow. Claims rest on primary reports, never on a review’s assertion, and a review cannot establish priority. Results from non-alcohol models and from MASH models are labelled as such in the sentence that carries them: they raise plausibility, and serve as liver-side benchmarks of what a drug class can do to a liver, but they are not evidence in an alcohol context. Registry records support design, endpoints and status only, with the date of checking. Where two studies of one design class contradict each other, the smaller does not carry the claim; where two studies of different design classes disagree, the claim may stand, and the sentence states which design it rests on. And a cell-type assignment is only as good as its reagent, a problem Section 3.1 meets at the GLP-1 receptor.

Human genetics is weighed above animal mechanism for an empirical reason. Genetic support roughly doubles the estimated success probability across the development pipeline, an advantage that grows with confidence in the causal gene but not with effect size—a general-pipeline result making an instrument’s validity, not its effect size, what must be appraised [32]. Both sides are nonetheless always reported together, because causal confidence comes from triangulating approaches whose biases differ, not from accumulating ones that share a bias [33].

The candidates come from PubMed searches run through the E-utilities and closed on 17 October 2026, in seven query families: the axis routes, the endpoint and model literature, and one for each of the five target blocks that Section 3, Section 4, Section 5 and Section 6 take in turn. Each target block was crossed in the title/abstract field with a drinking block, (alcohol OR ethanol OR drinking OR craving)[tiab] together with the MeSH terms Alcoholism and Alcohol Drinking, and separately with a liver block, (liver OR hepatic OR steatosis OR fibrosis OR hepatitis)[tiab] together with the MeSH term Liver Diseases, Alcoholic, so that each side of a candidate was retrieved on its own and neither side inherited the other’s hits.

The full Boolean strings for every block, with the number of primary records each retrieved, are given in Supplementary Methods S1 so that the search can be re-run exactly.

Screening ran in two passes. Titles and abstracts were read for whether a named molecule—a receptor, ligand, transporter or enzyme—had been manipulated or measured alongside a drinking or a liver readout; the records that survived were then read in full for the cell type, the model, the direction of effect and, for a trial, the endpoint class. A candidate entered the matrix on four conditions: that it is a molecularly defined molecule, named as such rather than as a community phenotype; that it has at least associative evidence on both the behavioural/central and the hepatic side; that the evidence sits in a retrievable peer-reviewed primary report; and that the report is neither a preprint, a news item, a commentary nor an editorial, and carries no retraction or erratum touching the claim.

Three candidates the searches returned did not enter the matrix, and the reasons are worth stating because they are what the both-sides requirement means in practice. Proprotein convertase subtilisin/kexin type 9 (PCSK9) has primary hepatic evidence in an alcohol model, but the only retrieved source on the drinking side is a review proposing the molecule as an axis target; a review is not evidence here, so the candidate has one side and not two. Lipocalin-2 is the mirror image, with primary liver-side evidence in alcohol-related injury and no brain-side evidence retrieved at all. Ghrelin O-acyltransferase (GOAT) fails a different test: it is the enzyme that acylates ghrelin rather than a node of its own, and acylation is appraised inside the ghrelin target in Section 3.4, where it bears on drinking, so listing it separately would count one system twice. The records each of these three searches returned are listed in Supplementary Methods S1. ClinicalTrials.gov, read through the API in the same window, returned 1864 unique interventional records after de-duplication, 638 of them drug or biological trials in an alcohol condition begun in 2016 or later; the 64 that bear on one of the sixteen targets or on the liver and alcohol-use endpoint classes were verified field by field and are the census Section 2.3 and Table 1 use. This is a narrative review with a declared search, not a scoping review.

### 2.3. What a Dual-Endpoint Trial Must Measure

The strongest claim is available only where both organ sides are measured in the same participants; otherwise a two-organ molecule is indistinguishable from a single-organ one. Whether treating alcohol use changes liver-disease progression is untested rather than answered [38]; the recommended exposure measure is quantified, verified intake over three to six months [39]. PEth is the instrument that window implies, but it is not a neutral ruler. Its sensitivity at the ≥8 ng/mL threshold was 81.8% in an individual-participant meta-analysis whose reference standard is self-report, the field’s own limitation, and that sensitivity rose with haemoglobin and with indeterminate or advanced liver fibrosis in an adjusted, exploratory model [40]. The intake measure is therefore modified by the fibrosis the same trial measures, and by the anaemia of the cirrhosis it is measured in.

Measurement also fixes the population: adding PEth quadrupled MetALD and tripled ALD diagnoses among adults with imaging-defined steatotic liver disease, 15.9% of whom underreported drinking [41]. Nor are the two sides substitutes: in a prospective cohort at risk of ALD, PEth predicted decompensation independently of non-invasive fibrosis tests, its discrimination decaying from six months to two years while the fibrosis tests held [42]—independent prognostic information means different quantities. Timing follows: PEth separated a GLP-1R agonist from placebo only at week 26 [36].

The two endpoint classes differ in what they measure. PEth is a phospholipid that forms only when ethanol is present, so its concentration tracks exposure. The Enhanced Liver Fibrosis (ELF) score is a serum index validated against biopsy for staging, with areas under the curve of at least 0.90 [43], and never validated for responsiveness to treatment-induced change. Responsiveness is the property a trial endpoint needs, which is why an ELF-primary trial returns a biomarker result rather than a histological one. Its insensitivity to drinking status is a virtue, not the limitation it appears to be: ELF was essentially stable across one to three months of abstinence or resumed drinking in a 35-patient pilot, so a trial-period change is no abstinence artefact [44]. That pilot had only eight and 13 resumers by days 30 and 90, however, and a significant first-week rise, 9.24 to 9.74, which makes baseline timing matter in detoxification [44]. Elastography, the readout trials pair with ELF, matches it for staging and holds discrimination to two years [42,43], but stiffness is inflated by steatohepatitis and recent intake, so where drinking falls it falls for the wrong reason—a further argument for co-primary endpoints. On the alcohol side, a two-level reduction in World Health Organization risk-drinking levels is a qualified primary endpoint [45]—a regulatory fact, not target engagement. It is also the primary endpoint of the largest trial the field has run on a dual-target agent: a recruiting phase 3 of semaglutide in AUD, 622 participants estimated, with no liver endpoint (NCT07218354). The two endpoint classes are not independent either: fibrosis screening with counselling in AUD was followed by 45% abstinence at six months [46].

Of 64 ClinicalTrials.gov records verified on 17 October 2026, one makes a liver outcome and an alcohol-use outcome both primary: the IL-22 combination trial, whose agent is the only one of the sixteen with published human data of its own (NCT07060638) [47]. Eight further records pair a liver outcome with an alcohol-side readout, with exactly one side primary; six of those eight count as pairing the two endpoint classes and two do not, because an alcohol-use outcome is counted here as consumption, abstinence or an intake biomarker and not as the craving, cue-reactivity or withdrawal-severity readouts that show target engagement alone—of the two set aside, one keeps its liver outcome secondary behind a cue-elicited-craving primary and the other carries liver enzymes as secondary endpoints with no consumption endpoint at all. One plus six is seven records in all that pair a liver outcome with an alcohol-use outcome, and none of the 64 pairs histology with an objectively measured intake endpoint as co-primaries (Table 1). Co-primary endpoints are therefore an instrument of molecular validation before a regulatory one: without them a dual mechanism reads as a single-organ effect, and single-side programmes like canakinumab in AH [10] cannot separate a failed target from an untested one. The incretin and hepatokine programmes have all chosen biomarker liver endpoints—ELF, PRO-C3, elastography—so none of them can return a clinical liver result whatever it finds. The two that chose endpoints capable of one are clinical decompensation in the IL-22 combination trial and histology in the canakinumab trial: the ones a dual-target reading most needs, and the smallest. And since measurement feeds back on behaviour, a dual-endpoint trial must prespecify what participants learn about their fibrosis.

## 3. Incretin and Appetite-Regulating Receptors

### 3.1. GLP-1R: From Septal Circuits to Hepatic Ethanol Handling

**Figure 2:**
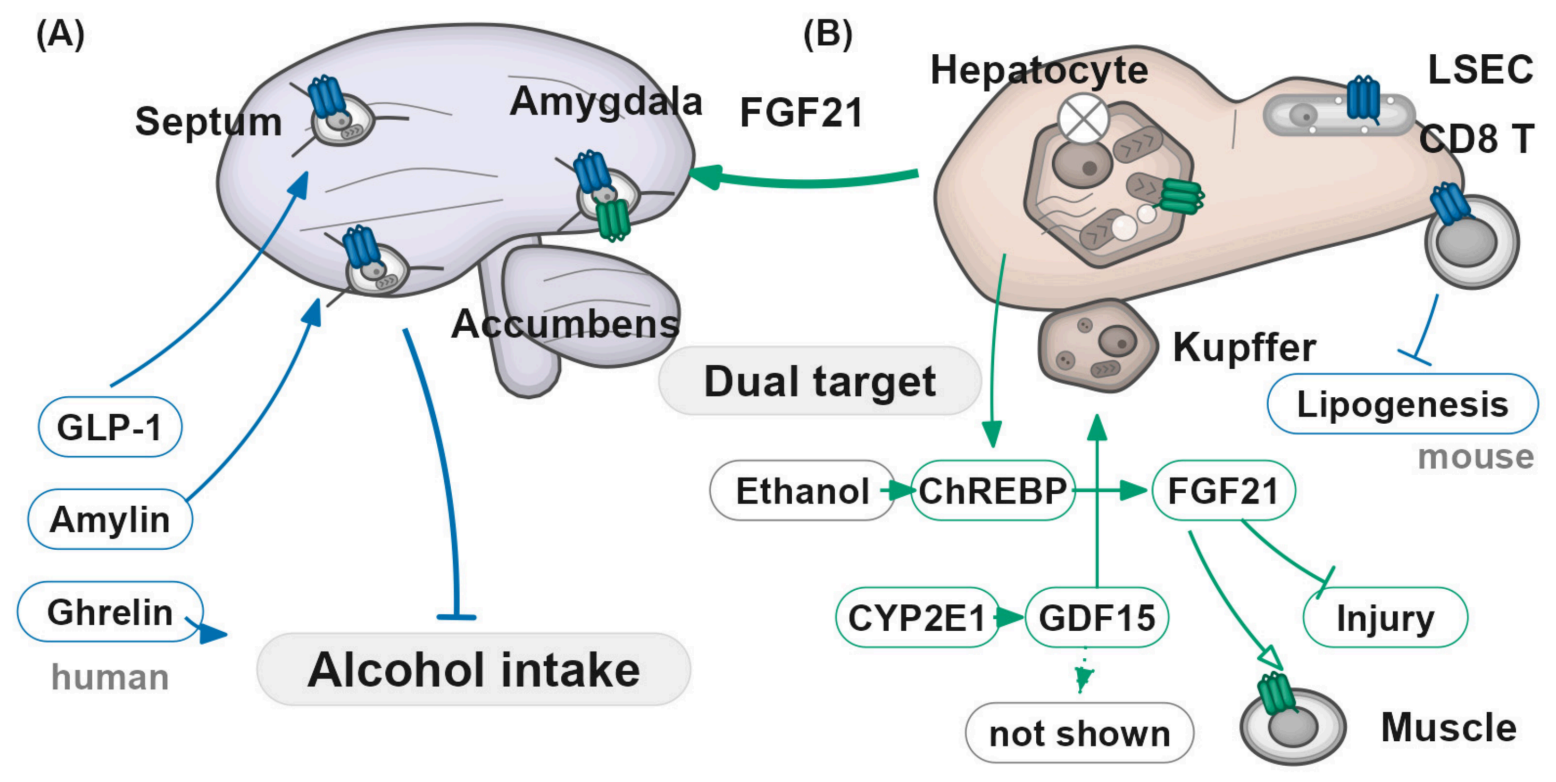
The molecules that set alcohol intake in the brain are the same molecules that act on identified liver cells. (**A**) Two brain nodes carry the behavioural arm: glucagon-like peptide-1 receptor agonism in the lateral septum lowers drinking, and fibroblast growth factor 21 acts through β-Klotho-expressing reward neurons, so engagement of either lowers alcohol intake. Centre: the four shared molecules, each named once. A flat head marks an arm that lowers drinking and a plain head one that raises it; ghrelin is the arm that raises, since intravenous ghrelin raises self-administered alcohol in humans. GDF15 carries no arrow: its receptor GFRAL sits in the brainstem, but no alcohol readout has been reported, which the dotted arrow and “Not shown” record. (**B**) The same families act on the liver. Hepatic GLP-1 receptor is expressed on liver sinusoidal endothelial cells (LSEC) and CD8^+^ T cells, not on hepatocytes (⊗), and agonism lowers hepatic lipogenesis; ethanol induces hepatocyte FGF21 and perivenous hepatocyte GDF15, which signals to Kupffer cells; the same FGF21 acts on muscle satellite cells in the opposite therapeutic direction (“Discordant”); and no hepatic amylin-receptor action has been shown in any alcohol model (⊗). Conventions: blue, incretin and appetite receptors; green, hepatokines. Solid arrow, activation; flat bar, inhibition; dashed, non-alcohol model; dotted, not yet shown; ⊗, nothing shown in that slot. Italic *mouse* and *human* mark species only where the mechanism could be misread. *Abbreviations:* CD8, cluster of differentiation 8; FGF21, fibroblast growth factor 21; GDF15, growth differentiation factor 15; GFRAL, GDNF-family receptor α-like, GDNF being glial cell line-derived neurotrophic factor; GLP-1, glucagon-like peptide-1. The evidence, species, model and citation for every element of (**A**,**B**) are given in Table S1 and in Section 3, Section 4, Section 5 and Section 6.

GLP-1R is the best-resolved candidate on the axis, and its drinking mechanism is localised to a defined circuit. In mice, systemic liraglutide lowered alcohol intake and blunted ethanol-evoked accumbens dopamine release only where GLP-1R was present in the dorsal lateral septum, acting through an inhibitory dorsal-septal GLP-1R to ventral-septal Esr1^+^ projection; inactivating the dorsal-septal GLP-1R neurons raised consumption and abolished the drug effect [12]. Septal signalling is bidirectionally sufficient and necessary in mice and rats: intra-septal exendin-4 reduced intake dose-dependently without changing food or water consumption, local receptor inhibition increased intake, and slice recordings traced the effect to GABA-A-receptor-dependent depression of septal transmission [48]. That is the cleanest available dissociation from general consummatory behaviour, though the expression–intake correlation held in males only [48]. This does not generalise. Semaglutide reduced binge-like and dependence-induced drinking in both sexes of two species and raised inhibitory transmission in central amygdala and infralimbic neurons, yet it also lowered intake of other caloric and non-caloric solutions, and that effect on transmission was absent in alcohol-dependent rats [49]. The node is septal–mesolimbic, its pharmacology state-dependent and only partly reinforcer-selective (Figure 2).

The first hepatic question is which cell carries the receptor. Spatial and single-cell profiling in mouse MASH models—a labelled liver-side benchmark—placed hepatic *Glp1r* on pericentral liver sinusoidal endothelial cells and CD8^+^ T cells rather than on hepatocytes, and endothelial *Glp1r* deletion or knockdown largely abolished semaglutide’s hepatic benefit while weight loss was preserved [11]. Why immunodetection could not settle this earlier is a reagent problem: a cell-type assignment is only as good as its antiserum, and none of three widely used antisera against the GLP-1 receptor could be validated, while the receptor’s coding sequence was undetectable in isolated hepatocytes and the hepatic effect proved indirect, central as well as peripheral dosing lowering plasma triglycerides [50]. In alcohol models the hepatic arm is pharmacological, not cell-resolved: in rats on a Lieber–DeCarli ethanol diet, exendin-4 lowered serum alanine aminotransferase and hepatic triglyceride and raised adiponectin without changing body-weight gain [51]. In mice on an ethanol diet, semaglutide reduced steatosis with suppressed lipogenic (*Acaca*, *Srebf1*, *Fasn*), redox (*Nfe2l2*, encoding the antioxidant regulator NRF2, and *Cybb*) and inflammatory (*Mpo*, *Il6*, *Il12*) transcripts and less 4-hydroxynonenal adduct formation [52]. That was on n = 3–4 per group, and the drug also lowered control-diet intake and body weight, so caloric restriction is not excluded as the route to less steatosis [52]. Semaglutide also lowered hepatic CYP2E1 and raised blood ethanol after gavage and intraperitoneal dosing, so altered ethanol handling is a pharmacokinetic consequence, not a benefit [52].

The human layer is translational, not interventional. Multi-ancestry drug-target Mendelian randomisation at the *GLP1R* and *GIPR* loci linked proxied agonism to less binge drinking, with both loci independently protective, null estimates for other substances, and lower MASLD risk and alanine aminotransferase—a liver-side benchmark, from instruments proxying body-mass and glycaemic lowering [53]. A target-trial emulation in veterans with harmful alcohol use—pharmaco-epidemiology, which cannot substitute for a trial—reported a lower composite liver outcome and lower odds of a positive drinking screen in the same people [54]. The same emulation reported an all-cause mortality hazard ratio of 0.43, larger than any plausible liver-mediated effect and a mark of residual healthy-user confounding [54]. In another observational cohort the weekly-intake reduction lost significance after adjustment for weight change, leaving separability from weight loss unsettled [55].

The randomised record of the class belongs in one passage, and it separates by endpoint rather than by trial. The first randomised trial in AUD—48 non-treatment-seeking participants over 9 weeks, reaching 1.0 mg for one week only—reduced laboratory alcohol self-administration and drinks per drinking day, with drinks per calendar day unchanged [56]. The largest and longest positive trial cut heavy drinking days by 13.7 percentage points more than placebo at 26 weeks in AUD with obesity [34]. Oral semaglutide missed its cue-elicited-craving primary endpoint and drinks per day while reducing heavy drinking days and drinks per drinking day [57]. Exenatide was formally negative on heavy drinking days [35], its secondary analysis detecting a PEth-confirmed fall only at week 26 [36]. Pooled, the randomised evidence is non-significant for consumption, drinks per drinking day and craving, from a search closing before the largest positive trial [58]. Against it stands a second synthesis, mixing randomised with observational designs, that reports an implausible 7.81-point AUDIT reduction with 87.5% heterogeneity [59]; the disagreement between the two is the content. No alcohol-population trial has yet reported a liver endpoint: liver enzymes and the fibrosis-4 index were prespecified secondaries only (NCT05895643). The proof that receptor engagement moves liver histology is a labelled MASH benchmark, in which an 8.5-percentage-point weight difference leaves a weight-independent hepatic mechanism unidentifiable [60] (Table 1).

The two sides of GLP-1R are therefore unequal (Table S1). The drinking side reaches randomised human trials; the liver side rests on the alcohol-model manipulations above and not on the human layer, which is contested—one target-trial emulation positive for both a liver composite and an alcohol-use outcome in the same cohort, against a larger active-comparator cohort in an alcohol population that was null. Both organs do move in the desired direction under the same strategy, semaglutide in human randomised trials and semaglutide in ethanol-fed mice, but in different species and in separate experiments, and never both after one manipulation. Agents with an alcohol indication are already in phase 2 and phase 3. The in-journal review of this class covers ALD in a dedicated section built on two rodent NIAAA-model studies and database analyses [22]. Of the additions set out in Section 1, this subsection supplies the cell-resolved hepatic arm, the septal circuit, locus-separating genetics, the randomised series with its negative and failed-primary results, and weight-independence. The most informative missing experiment is endothelial *Glp1r* deletion in a chronic-plus-binge or MetALD model.

### 3.2. GIP/GLP-1 Co-Agonism and the Broader Incretin Class

Adding glucose-dependent insulinotropic polypeptide (GIP) receptor agonism engages the same forebrain node. In rodents, the GLP-1R/GIP receptor co-agonist tirzepatide attenuated alcohol reward (locomotion, place preference and accumbens dopamine release), reduced voluntary intake dose-dependently and prevented binge-like and relapse-like drinking without tolerance, with sustained synaptic depression and altered histone regulatory proteins in the lateral septum [61]. The hepatic readout in the same animals was triglyceride content, in a metabolic rather than an alcohol-injury context, and no receptor-selective arm separates the two receptors [61]. Human evidence is genetic and pharmaco-epidemiological. The *GIPR* instrument was protective independently of *GLP1R* [53]. In four parallel target-trial emulations in type 2 diabetes, tirzepatide and semaglutide were associated with lower incident AUD, whereas liraglutide and dulaglutide were not, and tirzepatide also beat liraglutide head-to-head—a gradient tracking potency and weight loss as much as receptor profile [62]. A phase 2 trial of tirzepatide in MetALD with AUD is recruiting (NCT07046819). The two sides of GIPR are therefore unequal: the drinking side rests on manipulation in rodents, while the liver side has only that metabolic readout, so nothing yet shows one manipulation moving intake and alcohol-related liver injury together (Table S1). The most informative missing experiment is a receptor-selective comparison on intake and liver histology in one model.

### 3.3. Calcitonin–Amylin Receptor Signalling

The clinical amylin analogue is not receptor-selective, which constrains every inference from it. Cryo-electron microscopy showed cagrilintide activating both the amylin receptor AMY1R—the calcitonin receptor with receptor activity-modifying protein 1 (RAMP1)—and the bare calcitonin receptor, through a shared bypass binding mode [63]. Rodent drinking pharmacology is bidirectional and only partly reinforcer-selective: salmon calcitonin reduced intake, relapse-like drinking and operant self-administration in alcohol-preferring rats but not chocolate self-administration, while also lowering food intake, and the antagonist AC187 raised short-term intake without affecting food intake [64]. Durability is the limit, and it tracks selectivity: a selective long-acting agonist lowered intake initially in both sexes, but the effect ceased and intake increased in males while food intake and weight kept falling [65], whereas the non-selective agonist studies report no comparable loss of effect [64].

No hepatic amylin-receptor action has been shown in any alcohol model, so no liver endpoint of the ALD trial may be credited to cagrilintide ahead of the result, although a cagrilintide-monotherapy arm is one of its seven (NCT06409130). For the agent alone the only human hepatic datum is unchanged exposure across mild to severe hepatic impairment in a phase 1 study—pharmacokinetic safety, not efficacy [66]. In combination with semaglutide, cagrilintide did not improve fibrosis at 52 weeks in a MASH benchmark trial [67]. Calcitonin–amylin signalling therefore has a drinking side resting on manipulation in rodents and, in alcohol, an empty liver side, so nothing connects the two (Table S1). The most informative missing experiment is a selective agonist given to ethanol-fed animals with histological endpoints.

### 3.4. Ghrelin–GHSR and LEAP2

The ghrelin system is the methodological template for dual appraisal on this axis, the first molecule appraised across both AUD and ALD [20]. Intravenous ghrelin increased alcohol infusions by about 25% in a human laboratory paradigm, and raised the amygdala alcohol signal, in alcohol-dependent heavy drinkers [68]. Agonism therefore worsens drinking, and GHSR must be blocked rather than stimulated. Acylation, not abundance, sets the sign: des-acyl ghrelin, the predominant circulating form, reduced drinking in male and female rats and blocked alcohol-evoked accumbens dopamine in male rodents, through an unidentified receptor [69]. The endogenous antagonist comes from the liver: third-ventricular liver-expressed antimicrobial peptide 2 (LEAP2) suppressed alcohol-induced locomotion and reward memory, attenuated accumbens dopamine and lowered intake in both sexes [70]. Serum LEAP2 did not track consumption in rats, however, so hepatic production is not itself a hepatic mechanism [70]. The hepatic arm is an intervention in an alcohol model, albeit indirect and with no hepatic cell type identified: GHSR blockade in ethanol-fed rats restored pancreatic insulin secretion, lowered serum free fatty acids and reduced hepatic fatty-acid uptake and synthesis, attenuating alcohol-related steatosis [71]. Receptor crosstalk closes the loop to Section 3.1: chronic ethanol raised GLP-1 and GLP-1R in wild-type but not GHSR-knockout rats, ghrelin promoted GHSR–GLP-1R heterodimerisation that blunted GLP-1 signalling in transfected cells, and the knockout rats stayed insulin-sensitive and resistant to ALD despite lower GLP-1 [72]. That account inherits the localisation uncertainty above.

In humans, a GHSR inverse agonist reduced neither cue-elicited craving nor cue reactivity in AUD, while its effect on food choice survived; but the trial was terminated for COVID-19 with 29 completers on a single-session cue-reactivity endpoint, so it cannot be read as a negative result [73]. Both sides therefore rest on manipulation—a human single-session laboratory paradigm on the drinking side, an animal intervention on the liver side—with no manipulation read out on both organs, and the one treatment-period trial establishes nothing either way (Table S1), though an agent has reached people with an alcohol indication. The most informative missing experiment is one GHSR ligand tested on intake and on liver histology in the same animals.

### 3.5. Critical Appraisal of the Incretin Family

Mechanistically the family is asymmetric: the brain-side manipulations separating alcohol from food acted locally, whereas every hepatic result in an alcohol model is steatosis-level in male rodents, without fibrosis or cell-type resolution [51,52]. The human evidence divides the same way. Pooled randomised data were non-significant for consumption, drinks per drinking day and craving in a synthesis whose search closed on 3 May 2025, before the largest positive trial and before the oral-semaglutide trial [58]. Against approved AUD pharmacotherapy, one-year relapse fell while decompensated cirrhosis only trended lower and mortality was unchanged, in an observational cohort that excluded patients with advanced liver disease within one year—removing the population in which a hepatic effect would matter most [74]. The largest active-comparator cohort in an alcohol population is pharmaco-epidemiology, and it is null: GLP-1 receptor agonist initiation was not associated with less progression to cirrhosis (odds ratio 1.10, 95% CI 0.97–1.26) or fewer alcohol-related admissions [75]. Its population was defined as AUD with type 2 diabetes rather than as established alcohol-related cirrhosis.

The sarcopenia objection is answered reassuringly only in a cholestatic mouse model, not in alcohol [76]. No study has yet reported hepatic or nutritional safety outcomes of incretin therapy in a population defined by established alcohol-related cirrhosis. The one registered trial in ALD excludes decompensation, liver stiffness ≥ 25 kPa, grade ≥ 2 varices and lean patients, so it cannot answer that either. That trial completed on 12 January 2026 with change in the ELF score—a biomarker, not histology—as its only primary endpoint, and no results had been published as of 17 October 2026 (NCT06409130).

## 4. Hepatokines as Brain-Acting Drugs

### 4.1. FGF21–β-Klotho

FGF21 acts only on cells expressing the co-receptor β-Klotho (gene *KLB*), which is required for FGF21 to regulate alcohol preference; brain-specific β-Klotho deletion increases alcohol drinking in mice, placing the behavioural arm inside the central nervous system (CNS) [77]. The circuit is projection-specific: FGF21 suppresses alcohol consumption through KLB-expressing basolateral amygdala neurons in mice, and an FGF21 analogue halved alcohol intake in alcohol-preferring non-human primates [14]. The limit is the only loss-of-function test outside the mouse: in the first FGF21-knockout rat line, endogenous FGF21 did not regulate drinking [78]. The arm is therefore established for analogue pharmacology and brain-restricted β-Klotho loss, not for the endogenous hormone across species (Figure 2).

Ethanol induces the hepatokine through two of its own catabolites: in mice and cell-based assays, glycerol-3-phosphate binds and activates the carbohydrate-response-element-binding protein (ChREBP) at the *Fgf21* promoter, while acetyl-CoA drives the histone acetylation ChREBP recruitment requires [13]. Hepatic FGF21 is protective, with the cell type identified: hepatocyte-specific FGF21 deficiency aggravates steatosis, inflammation and acute-on-chronic injury in chronic-plus-binge ethanol-fed mice, in a model in which FGF21 induction itself runs through SIRT1 [79]. The direction is not uniform. Whole-body *Fgf21*-knockout mice are instead protected from alcohol-induced fatty liver, because FGF21 is required for the systemic catecholamine surge that drives adipose lipolysis and fatty-acid delivery to the liver [80]. In knockout rats on a Gubra-Amylin–NASH diet plus alcohol—a MASH-like diet model, not an alcohol model—the same loss raised transaminases and hepatic triglyceride but left every histological endpoint unchanged [78]. For the alcohol results the reconciliation is which compartment supplies the lipid: adipose-derived fatty acid, removed by whole-body loss, against hepatocyte-intrinsic protection, removed by hepatocyte-specific loss [79,80]. A separate dissection, in MASH and benchmark-labelled, separates organs rather than tissues: FGF21 signalling to central glutamatergic neurons raises hepatic sympathetic drive, suppresses lipogenesis and reverses fibrosis, whereas hepatocyte-directed signalling is necessary and sufficient only for lowering hepatic cholesterol [81].

Human genetics is the strongest single piece of dual evidence here. *KLB* variation (rs11940694) is associated with alcohol consumption at genome-wide significance in more than 105,000 individuals of European ancestry [77]. Mendelian randomisation links genetically higher FGF21 to less problematic alcohol use and to lower risk of ALD, implicating hippocampal FGFR3 and basal-ganglia *KLB* expression [82]. An independent instrument, the common *FGF21* L174P missense variant, reproduces that association for consumption and AUD with supporting colocalisation, but uses liver fat and cirrhosis only as positive controls rather than an ALD endpoint, so the two papers are not two independent ALD results [83]. Three caveats travel with it. The first two are methodological: the data are European-ancestry under standard Mendelian-randomisation assumptions, and the ALD estimate is the weakest in its paper while the problematic-use signal is the weakest in the replication [82,83]. The third is substantive: mediation attributes the effect entirely to behavioural pathways and a raised basal metabolic rate, so the genetics support a brain-mediated liver benefit rather than an independent hepatic one [82,83]. The circulating biomarker points the other way, being highest in moderate-to-severe AH and discriminating it from decompensated alcohol-related cirrhosis [84]—resistance or feedback failure rather than protection (Table S1).

The clinical-stage agents are analogues. NNC0194-0499 is reported as the FGF21 analogue zalfermin, albumin-binding for once-weekly dosing [85]—an attributed inference, since the registry record states no molecular target. Efruxifermin improved fibrosis by ≥1 stage without worsening steatohepatitis in a phase 2b MASH trial [86]—a liver-side benchmark, which cannot stand in for evidence in an alcohol context, though it is what has brought an FGF21 analogue into people. Zalfermin with semaglutide, the only FGF21-analogue combination tested there, did not improve fibrosis at 52 weeks, while semaglutide alone was nominally significant [67]. In ALD, the seven-arm phase 2 trial, including NNC0194-0499 (NCT06409130), has completed without reported results (Section 3.5, Table 1).

For FGF21 the strongest evidence on either side is human and genetic rather than interventional, and the two sides cannot be read as pointing the same way: the genetic liver benefit is brain-mediated, the circulating hormone is highest in the most severe disease, and the muscle arm of Section 4.3 moves a second organ the wrong way (Table S1). No trial result in an alcohol population exists, and the analogue the one ALD trial contains is identified as an FGF21 agent only by inference, so the class’s unambiguous clinical presence is in other indications. The most informative missing experiment is a β-Klotho-dependent analogue tested on voluntary intake and liver histology in the same alcohol-fed animals, with the hepatocyte and central arms separated.

### 4.2. GDF15–GFRAL

Growth differentiation factor 15 (GDF15) is the second hepatokine with a defined central receptor, its brain side anatomically precise but behaviourally untested for alcohol. The receptor, GDNF-family receptor α-like (GFRAL), is restricted in the mouse brainstem to the area postrema and nucleus tractus solitarius, from where GDF15 recruits the parabrachial nucleus and central amygdala [87]. *Gfral*-knockout mice are hyperphagic under stress and resistant to chemotherapy-induced anorexia—none of it with an alcohol readout [87] (Figure 2).

The hepatic evidence is loss-of-function. Alcohol directly induces GDF15 in primary mouse hepatocytes, and *Gdf15*-knockout mice accumulate TNF-α-producing and activated CD4^+^ and CD8^+^ T cells, infiltrating monocytes and neutrophils, with worse inflammation [88]. That study used chronic alcohol and carbon tetrachloride together, so the two arms’ contributions to the cell counts are unresolved [88]. The induction route has been resolved in mice, with corroborating human early-ALD tissue: gut-derived catecholamines act on β2-adrenergic receptors on perivenous hepatocytes, driving mitochondrial CYP2E1 translocation and hepatic GDF15, which then raises the same receptor on inflammatory Kupffer cells so that catecholamine signalling kills those cells [89]. Ablating hepatic *Gdf15* reduced that apoptosis and worsened alcohol-related inflammation [89]. Whether that benefit is merely weight loss is answered in a MASH model, benchmark-labelled: deletion of *Gdf15* or *Gfral* selectively worsened hepatic inflammation and fibrosis, and recombinant GDF15 outperformed weight-matched caloric restriction through a GFRAL-dependent neuroendocrine glucocorticoid route [90]. That route raises its own unaddressed safety question in cirrhosis, and it sits against the aversion-based account in Section 4.3 [90].

In humans, one week of binge drinking with fast food raised fasting GDF15 alongside FGF21, aspartate aminotransferase and liver stiffness in 14 young men—a confounded exposure that nonetheless dates the induction to days [91]. In AH, GDF15 is expressed in senescent hepatocytes as part of a senescence-associated secretory phenotype and correlates with severity markers, and plasma GDF15 predicted corticosteroid response and 90-day mortality in two independent cohorts [92]. Human genetic evidence linking GDF15 or GFRAL variation to alcohol consumption has not been reported in a peer-reviewed study, and no agent engaging this axis has entered a trial with an alcohol indication.

The two sides of GDF15–GFRAL are therefore unequal (Table S1). The drinking side is association alone—the receptor’s anatomical restriction and the human GDF15 rise described above—because no alcohol-relevant manipulation of the receptor exists [87,91]. The liver side has loss-of-function manipulation with the cell type identified in alcohol models [88,89], while the prognostic human marker points against the protective reading rather than supporting it [92]. No manipulation has been read out on both organs, and no clinical-stage agent engages this axis. The most informative missing experiment is GFRAL-dependent GDF15 agonism tested on voluntary intake and liver histology in the same animals.

### 4.3. Critical Appraisal: Can One Hepatokine Axis Be Both the Signal and the Drug?

For GDF15 the therapeutic and the adverse effect are one mechanism: it causes emesis in an emetic species and nausea- and malaise-like behaviour in mice and rats, so its anorexia is produced through aversive rather than homeostatic circuitry [93]. An intake endpoint alone cannot distinguish an agent that devalues alcohol from one that makes the animal unwell. For FGF21 the muscle risk is mechanistic, not hypothetical: in decompensated cirrhosis, liver-derived FGF21 acts on β-Klotho on muscle satellite cells to raise PTEN, inhibit PI3K/Akt and block myogenesis, and neutralising FGF21 or deleting *Klb* in satellite cells reversed the sarcopenia [94]. That muscle arm is the reason FGF21’s two sides cannot be read as pointing the same way, in a population already prone to muscle loss—though that cohort’s aetiology is not alcohol-specified [94]. Skeletal safety under sustained receptor agonism is unaddressed rather than reassured. FGF21 nevertheless remains the cleanest test of the argument: the only target here with human genetic triangulation replicated for the drinking phenotype, a preclinical mechanism that agrees once the compartments are separated, and a published trial result—negative, and in MASH rather than alcohol [67].

## 5. Glutamatergic Signalling from Synapse to Sinusoid

### 5.1. CNS Glutamate in Alcohol Use Disorder

The glutamatergic node with human in vivo data in AUD is mGluR5, whose two positron-emission tomography (PET) studies disagree in direction. Dynamic[^18^F]FPEB PET found availability 14% to 36% lower in mainly limbic regions of 16 recently abstinent alcohol-dependent participants than in 32 controls, lower availability tracking higher hair ethyl glucuronide, an objective index of recent drinking [95]. [^11^C]ABP688 PET in 14 non-smoking men abstinent for at least 25 days found the opposite: higher availability across temporal regions and most in the amygdala, where higher binding accompanied less temptation to drink [96]. Tracer, kinetic model, smoking status and time since the last drink all differ, so reading low availability as a state of recent drinking rather than a trait remains a hypothesis neither study can test.

The proposed glutamate-receptor mechanism of acamprosate does not survive direct testing. The drug interacted with neither N-methyl-D-aspartate receptors nor group I mGluRs, and in rats the sodium salt of N-acetylhomotaurinate was inactive in operant ethanol responding, cue-induced reinstatement and the alcohol deprivation effect [97]. Calcium salts reproduced its effects, placing the activity in the calcium moiety [97]. The brain side therefore rests on associative human imaging of unresolved direction; the most informative missing measurement is a within-subject PET series spanning active drinking and sustained abstinence.

### 5.2. Hepatic Glutamate Release onto mGluR5-Bearing Liver Cells

In male C57BL/6J mice, chronic ethanol feeding induced VGLUT3 (*Slc17a8*) and excitatory amino-acid transporter 2 (EAAT2, *Slc1a2*) in perivenous hepatocytes, and a superimposed binge triggered SNARE-dependent glutamate exocytosis onto mGluR5 on adjacent Kupffer cells, driving NOX2 (*Cybb*), reactive oxygen species, CXCL1 and IL-1β release and neutrophil recruitment [15]. Kupffer-cell-conditional deletion of *Grm5* or *Cybb* was protective, and hepatocyte ballooning narrowed the intercellular gap, making proximity part of the mechanism [15]. The human arm is correlative, plasma glutamate being higher in alcohol-related steatohepatitis and correlated with aminotransferases in non-cirrhotic patients [15].

Two further sources converge on the same receptor. In chronically ethanol-fed mice, impaired transsulfuration depletes glutathione and the compensatory cystine–glutamate antiporter xCT exports glutamate onto stellate-cell mGluR5, whose activation generates 2-arachidonoylglycerol acting on hepatocyte CB1, inhibiting mGluR5 or xCT attenuated alcohol-related steatosis [98]. In bone marrow, stromal xCT-derived glutamate stimulated natural-killer-cell mGluR5, whose interferon-γ raised the IL-1β decoy receptor IL-1R2 on monocytes that limited liver injury, so natural-killer-cell *Grm5* deletion worsened it [99]. Human methylation data point at the same transporter: an epigenome-wide association study of alcohol consumption in 8161 individuals identified *SLC7A11*, which encodes xCT, as its top target [100]. “Hepatic mGluR5” therefore denotes at least three recipient cells with partly opposite consequences: engagement is injurious on Kupffer and stellate cells but protective on natural killer cells, and no published experiment reconciles them [15,98,99]. A liver–brain glutamate “metabolic synapse” was proposed earlier, in a review by two of the present authors [21]; the hepatocyte-to-Kupffer-cell account differs in the cell pair, in vesicular rather than transporter-mediated release, and in its dependence on ballooning-generated proximity [15]. The liver side rests on cell-type-conditional genetics, the human tissue evidence being correlative (Table S1), and no agent has been tested against a liver endpoint in a drinking population.

### 5.3. Pharmacology Shared by Both Compartments

N-acetylcysteine engages the same nodes, blocking ethanol-induced VGLUT3 and EAAT2 induction in mouse hepatocytes [15]. It did not beat placebo for substance-craving across nine randomised trials in 623 participants, alcohol being the target substance in three (standardised mean difference 0.19, 95% CI −0.02 to 0.39), with 99% heterogeneity and detectable publication bias [101]. The only clinical-stage mGluR5 negative allosteric modulator result is in cocaine use disorder: mavoglurant reduced cocaine use in 68 adults, while its secondary, self-reported (Timeline Followback) alcohol endpoint did not reach significance (*p* = 0.072) [102]. The only mGluR5-directed compound given with alcohol in AUD, GET 73, was tolerated but altered neither cue-induced craving nor laboratory self-administration on its single-session readouts, and its mGluR5 selectivity is only putative [103]. A mavoglurant study in heavy drinkers is recruiting and the GET 73 bar-laboratory and spectroscopy study completed unreported (NCT06136195, NCT04831684; checked 17 October 2026) (Table 1). Neither compound can therefore establish mGluR5 as an alcohol target, and the drinking side remains associative.

Because mavoglurant is brain-penetrant it cannot test the hepatic arm alone, and no peripherally restricted mGluR5 ligand has been reported—whereas at CB1 peripheral restriction reaches both the liver and, through vagal afferents, drinking (Section 6.4) [18,104]. For mGluR5, the tool compound is the missing reagent.

### 5.4. GRP–GRPR: A Parallel Candidate That Points Both Ways

Gastrin-releasing peptide (GRP) and its receptor (GRPR) reach the same myeloid oxidase by another ligand. GRPR was high in human alcohol-related steatohepatitis liver, with ethanol-induced histone H3 lysine-27 acetylation driving GRP expression; in mice, global and myeloid-specific *Grpr* deletion and the antagonist RH-1402 reduced ethanol-induced injury, steatosis and neutrophil influx through IRF1–caspase-1 and NOX2-dependent arms [105]. The brain side exists and points the other way. In rats bombesin reduced alcohol intake, a bombesin-receptor antagonist blocked that reduction, and the antagonist alone initially increased drinking [106]. In male mice, accumbens-specific *Grpr* deletion increased motivation on a progressive-ratio task rewarded with food, with no alcohol readout [107]. Receptor activation reduces drinking whereas hepatic benefit requires blockade, so the two sides of GRP–GRPR call for opposite pharmacology (Table S1). Both rest on manipulation in animals, the drinking side on a bombesin-receptor-family ligand that is not GRPR-selective [106] and with no human data at all, and no clinical-stage agent exists. The most informative missing experiment is a GRPR antagonist tested on intake and liver histology in the same animals.

### 5.5. Limitations of Targeting Hepatic Glutamatergic Signalling

The hepatic mechanism comes from male mice on two weeks of forced ethanol feeding with a terminal binge—steatohepatitis, not fibrosis, where intake cannot be an endpoint [15]. Whether hepatic glutamate is signal or spillover is unresolved. Conditional deletion of EAAT2, the liver’s only excitatory amino-acid transporter, gives no baseline phenotype and has never been tested under a binge [108]. Simultaneous VGLUT3 release and EAAT2 uptake in one hepatocyte could also prevent receptor activation, a caveat raised in that study [15]. Two compartment controversies forbid a blanket block of hepatic mGluR5. Natural-killer-cell mGluR5 is anti-fibrotic, its loss aggravating fibrosis in carbon-tetrachloride and methionine–choline-deficient models, not alcohol [109]. And whole-body NOX2 loss worsens alcohol-related injury through impaired macrophage efferocytosis after four weeks of chronic feeding [110], where Kupffer-cell-conditional deletion protects under two weeks plus a binge [15]—compartment and exposure both differ. The family’s drinking side therefore remains associative while its liver side rests on cell-type-conditional manipulation, and because the sign of hepatic mGluR5 engagement reverses with the cell engaged the two sides cannot be read as pointing the same way (Table S1); the one clinical-stage mGluR5 negative allosteric modulator was developed for another indication. The most informative missing experiment is a hepatocyte-specific *Slc17a8* knockout under chronic-plus-binge ethanol.

## 6. Microbiome-Derived and Shared Neuroimmune Targets

### 6.1. Microbial Effectors with Dual Readouts

**Figure 3:**
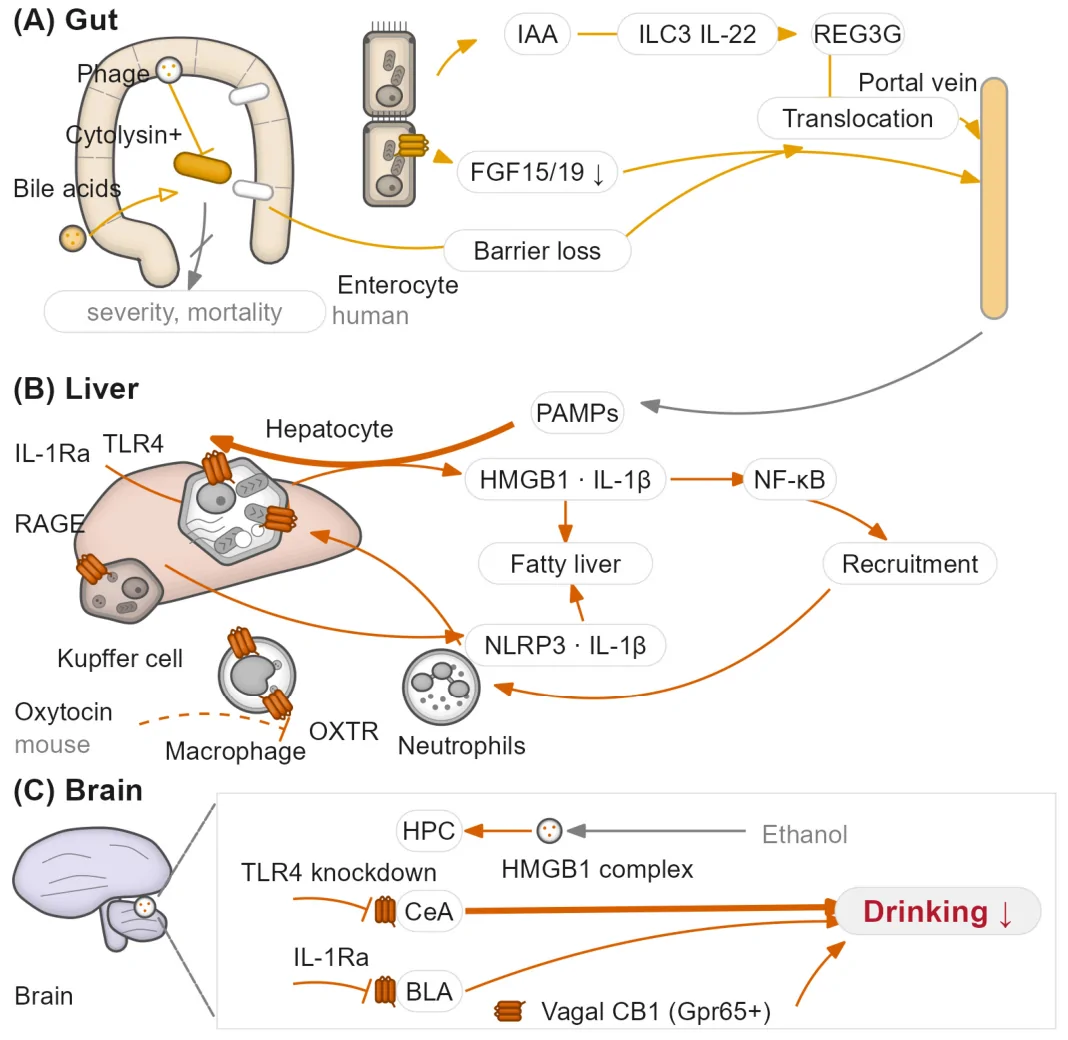
One innate-immune receptor module relaying gut microbial signals through the liver to the brain. Directional arrows indicate the direction of signalling between molecules, cells, and pathways. (**A**) Gut. A microbial aryl-hydrocarbon-receptor ligand raises innate-lymphoid-cell IL-22, which induces epithelial REG3G; cytolysin-positive *Enterococcus faecalis* translocates into the portal circulation, while the human carriage-and-survival association is drawn struck through because it failed independent replication; enterocyte FXR sets portal FGF15/19. (**B**) Liver. Portal signals act on hepatocyte TLR4 to drive fatty liver; the hepatocyte’s oxidised HMGB1–IL-1β complex signals through RAGE on Kupffer cells and myeloid-derived macrophages; inflammasome-matured IL-1β acting through IL-1R1 is required for injury; oxytocin acts on the macrophage oxytocin receptor to reduce fibrosis, dashed because its only evidence is a non-alcohol fibrosis model. (**C**) Brain. Knockdown of central-amygdala TLR4, infusion of an interleukin-1 receptor antagonist into the basolateral amygdala, and CB1 on Gpr65-positive vagal afferents each reduce drinking. Conventions: solid, shown in an alcohol model or in humans; flat bar, inhibition; dashed, non-alcohol model only; grey struck-through arrow, a failed human replication. BLA, basolateral amygdala; CB1, type 1 cannabinoid receptor; CeA, central nucleus of the amygdala; FGF, fibroblast growth factor; FXR, farnesoid X receptor; Gpr65, G-protein-coupled receptor 65; HMGB1, high-mobility group box 1; IL, interleukin; IL-1R1, interleukin-1 receptor type 1; IL-1Ra, interleukin-1 receptor antagonist; NLRP3, NOD-, LRR- and pyrin-domain-containing protein 3; RAGE, receptor for advanced glycation end-products; REG3G, regenerating islet-derived protein 3 gamma; TLR4, Toll-like receptor 4. The evidence, species, model and citation for every element of (**A**–**C**) are given in Table S1 and in Section 3, Section 4, Section 5 and Section 6.

Microbial effectors enter the axis as defined molecules, the best characterised a toxin (Figure 3): cytolysin, a two-subunit *Enterococcus faecalis* exotoxin, lyses hepatocytes [37]. Faecal cytolysin-positive *E. faecalis* tracked AH severity and mortality, and phages targeting cytolytic *E. faecalis* abolished ethanol-induced liver disease in microbiota-humanised mice [37]. The human half has not replicated: in a prospective Danish cohort of 28 patients with AH, only 10.7% were carriers and 180-day mortality did not differ [111]. The cohort is small and single-country, so regional carriage variation is as plausible as a false original claim; either way the human evidence is associative.

The best-resolved microbial mechanism is a complete ligand–receptor–peptide chain. Ethanol-associated dysbiosis lowers intestinal indole-3-acetic acid, an aryl-hydrocarbon-receptor ligand, reducing IL-22 from type 3 innate lymphoid cells and hence intestinal REG3G [16]. Supplying either the metabolite or IL-22-secreting engineered *Lactobacillus reuteri* prevented bacterial translocation and steatohepatitis in ethanol-fed mice, and that protection was lost in *Reg3g*-knockout mice, which identifies the required node [16]. It is also the one chain with human data for its agent. In 18 patients with moderate or severe AH, open-label recombinant IL-22 was tolerated to 45 µg/kg with falling MELD and aminotransferases and a day-7 Lille score ≤ 0.45 in 83%, against 6–56% in propensity-matched historical cohorts—a human result, though not a randomised one [47].

On the drinking side the evidence is interventional and community-level. In a randomised phase 1 trial in 20 men with alcohol-related cirrhosis, primary outcome 6-month safety, craving fell in 90% of recipients vs. 30% on placebo, with lower urinary ethyl glucuronide [112]. That is the only objective intake biomarker a microbiome intervention has moved, and it was a secondary, exploratory endpoint [112]. Pooled data from 444 patients with severe AH showed higher 28- and 90-day but not 6- or 12-month survival, the advantage confined to retrospective studies [113]. The cautionary case is sharper, the intervention having worked as designed. Seventeen days of inulin in 50 inpatients with AUD shifted microbiota composition without improving craving, depression or anxiety, though it raised a sociability score and serum brain-derived neurotrophic factor [114], and left aspartate and alanine aminotransferases and IL-18 higher than placebo [115]. No cytolysin-directed manipulation has a drinking readout—the interventions just described are community-level rather than target-directed—so cytolysin’s drinking side is empty, while its liver side rests on the phage manipulation and the human carriage data remain associative and unreplicated (Table S1). No cytolysin-directed agent has entered human study, and the registered phage and faecal-transplant records are not recruiting or unknown (NCT06750588, NCT05006430, NCT06496191; checked 17 October 2026). The most informative missing experiment is a prespecified multicentre replication with strain-level typing before further phage work.

### 6.2. Bile Acids and FXR/TGR5–FGF15/19 as Axis Signals

Bile acids are the axis’s own receptor ligands, and ethanol remodels them at source: enriched bacterial bile-salt hydrolase activity lowers enterocyte farnesoid X receptor (FXR) signalling and FGF15, de-repressing hepatic *Cyp7a1*; the intestine-restricted FXR agonist fexaramine and FGF19 each protected ethanol-fed mice [116]. The counter-result is from the same ligand. In female mice given supraphysiological adeno-associated-virus FGF19 with ethanol in a 10-day chronic-plus-binge model, sampled 9 h post-binge, hepatic *Il6*, *Tgfb1* and *Tnf* rose above ethanol alone, and secondary bile acids fell enough to permit overgrowth of Section 6.1’s pathobiont [117]. Model, sex, timing and overexpression separate them; neither need be wrong. Cell-type resolution decides it: global FXR deletion worsened injury, steatosis, inflammation and fibrosis at equal alcohol intake [118], whereas hepatocyte-specific deletion changed bile-acid and lipid gene expression but not histology [119]—the protective arm is intestinal, not hepatocellular. TGR5 deficiency worsened alcohol-induced injury in female mice through macrophage recruitment, reproduced by transferring the knockout microbiota to wild-type animals [120]. In humans, serum bile acids were 186.0 µM in AH, 64.5 in decompensated alcohol-related cirrhosis and 5.0 in health, FGF19 raised across two cohorts [121]. The brain arm is untested: no peer-reviewed study has manipulated a bile-acid receptor with an alcohol-intake endpoint, so the drinking side is empty. The one registered clinical test stopped for want of efficacy, not tolerability: the phase 2 FXR agonist INT-787 trial in severe AH, n = 67, a stopped programme rather than a reported negative result (NCT05639543; checked 17 October 2026). FXR and TGR5 therefore rest entirely on the liver, on cell-type-resolved manipulation with human biomarker support, and no manipulation has been read out on both organs (Table S1). An agent has nonetheless reached phase 2 in an alcohol indication, and its having been stopped bears on that agent rather than on access to the target. The most informative missing experiment is an intestine-restricted FXR agonist tested on histology and intake in one animal set.

### 6.3. Shared Innate-Immune Targets: TLR4–HMGB1 and IL-1β/NLRP3

Toll-like receptor (TLR) 4, high-mobility group box 1 (HMGB1) and IL-1β form one receptor module on both sides of the axis. In alcohol-preferring rats, central-amygdala but not ventral-pallidal TLR4 knockdown reduced binge drinking without altering sucrose intake [122]. In the same study, knocking down GABA-A α2 lowered TLR4 expression, so the two are co-regulated rather than independent [122]. In the liver the pool that matters is hepatocytic: hepatocyte-specific *Tlr4* deletion protected mice from chronic and binge alcohol injury, while myeloid deletion did not prevent fatty liver [123]. The ligands are physically coupled. HMGB1–IL-1β heterocomplexes potentiating IL-1 receptor signalling were raised in post-mortem human alcoholic hippocampus and induced in mouse brain by binge ethanol [124]. The hepatocyte-derived oxidised complex in AH livers signals through myeloid receptor for advanced glycation end-products (RAGE) rather than TLR4, and deleting RAGE there protected liver and gut barrier alike [125].

Hepatically the IL-1 arm is required: IL-1R1 with caspase-1/ASC maturation in Kupffer cells is necessary for alcohol-induced injury, and IL-1 receptor antagonist reverses it in mice [126]. Two agents built on that arm nevertheless failed in AH without showing IL-1 blockade ineffective. Anakinra, given with zinc in an active-comparator superiority design, was stopped early because prednisone gave higher 90-day survival—inferiority to steroids, not a property of the target [9]. Canakinumab gave no significant benefit on paired biopsies in 57 patients with 48 evaluable pairs, underpowered for the 16-percentage-point difference seen, while its *post hoc* adjusted analysis was nominally positive (*p* = 0.04) and hypothesis-generating [10] (Table 1).

The brain arm is now manipulable, and its results disagree. After a binge history that raised IL-1β in the basolateral, not the central, amygdala, IL-1 receptor antagonist infused there reduced binge-like drinking in male mice, with sucrose and locomotor controls [127]. That antagonist also blocks IL-1α at IL-1R1, so the ligand is not isolated [127]. Global deletion of *Nlrp3*, which encodes the NOD-, LRR- and pyrin-domain-containing protein 3 (NLRP3) inflammasome sensor, lowered binge intake while reducing glutamatergic drive onto striatal neurons—the junction with Section 5 [128]. Neither that deletion nor MCC950 blocked withdrawal escalation of two-bottle-choice drinking, and MCC950’s positive readout was anxiety rather than intake, so it does not confirm an effect on drinking [128]. And in constitutive *Il1r1*-knockout mice, intake and preference were unchanged across three consumption tests [129]. The brain evidence names sites, not cell types.

The two hepatic trial failures are therefore best read as failures of timing (Section 7), compartment and redundancy rather than of the target. A systemic blocker does not selectively reach the compartments engaged, and one node of a redundant module can be lost without moving the system. Both arms of the TLR4–HMGB1–RAGE module rest on manipulation in animals, with no manipulation read out on both organs (Table S1); its only clinical-stage programme is a phase 2 of the TLR4 antagonist TAK-242 in severe AH and acute-on-chronic liver failure (NCT06890039). IL-1β/NLRP3 stands likewise on manipulation on both sides, again never on both organs after one manipulation (Table S1). Its drinking-side manipulation survives a contradicting null because the two disagree across design classes rather than within one: the positive is acute, anatomically restricted antagonism of IL-1R1 in the basolateral amygdala, the null a constitutive germline deletion of the same receptor. Neither hepatic trial can settle the target—one underpowered on a histological endpoint, the other an active-comparator design against prednisone with zinc co-administered—so the liver side still rests on mechanism rather than on a trial. The most informative missing experiment is a cell-type-conditional brain IL-1R1 deletion read out on voluntary intake.

### 6.4. PDE4, CB1 and the Oxytocin Receptor: Three Partially Dual Targets

Phosphodiesterase (PDE) 4 degrades cyclic AMP in both compartments. Apremilast reduced binge-like and dependence-related drinking across mouse genetic backgrounds, apparently by raising nucleus-accumbens activity, and reduced drinking in a phase 2a study in non-treatment-seeking adults [130]. Ibudilast, by contrast, changed neither heavy drinking days nor any peripheral inflammatory marker over 12 weeks in 102 treatment-seeking adults [131]—non-engagement being an inference, peripheral markers a weak proxy for central engagement. A second molecule carries the dual readout: a 5-azaindazole lead reduced alcohol intake and alleviated alcohol-related liver injury in the chronic-plus-binge model, less emetogenic than roflumilast or apremilast and improving alcohol-induced sedation and motor impairment [132]. CB1 makes the compartment argument. Ethanol induces stellate-cell 2-arachidonoylglycerol acting on hepatocyte CB1, whose conditional knockouts are protected [133], and peripherally restricted antagonism is efficacious against alcohol-induced steatosis in mice [104]. Its anti-drinking effect requires CB1 on Gpr65^+^ nodose-ganglion vagal afferents rather than brain neurons [18], so both arms are reachable without central exposure. The oxytocin receptor diverges most: intranasal oxytocin had no effect on heavy drinking days, craving or mood in 100 adults [134], while its liver arm—fibrosis reversal through receptors on myeloid-derived hepatic macrophages, not stellate cells—rests on carbon tetrachloride and bile-duct-ligation models, not alcohol [135].

PDE4 reaches a human trial on the drinking side and animal manipulation on the liver side, and both organs move in the desired direction under one preclinical compound series, in separate experiments rather than in the same animals—though apremilast, the molecule taken into people, has never been given a liver endpoint (Table S1). CB1 rests on manipulation on both sides, and one peripherally restricted strategy reaches both arms in animals, again in separate experiments, but no clinical-stage antagonist exists (Table S1). The oxytocin receptor has a negative trial on the drinking side and nothing on the liver side in alcohol at all, its only liver evidence coming from non-alcohol models, and its uncertain central exposure makes that trial a verdict on route and agent rather than on the receptor (Table S1). These three need a subtype-selective PDE4 inhibitor and a myeloid oxytocin-receptor manipulation tested on intake and histology in the same animals.

### 6.5. Critical Appraisal

Three limits keep this layer’s evidence below its mechanistic detail (Table S1). Alcohol consumption frequency is among the strongest sources of gut-microbiota variance and differs between cases and controls, so matching on it shrinks apparent disease associations [136]—also the most charitable reading of the cytolysin non-replication [111]. A transferable community phenotype is not a druggable molecule, a predicted-direction microbiota shift need move neither organ, and a redundant module survives the loss of one node [113,114,115,131]. The opposed directions at FGF19 and the oxytocin receptor are informative, not disqualifying, but no target here should enter phase 2 on mechanism alone.

## 7. Discussion: Translating Dual Targets

Read across Table S1, the dual-target premise survives in narrower form than the literature suggests, and two standards have to be kept apart to say how narrow. A target is supported on both sides when evidence exists on the drinking side and on the hepatic side, whatever experiments the two came from. A target is demonstrated to act on both organs in the same experimental system only when one manipulation moved a drinking readout and a liver readout in one set of animals or participants. No target in this census meets the second standard, so every agreement between the two sides reported here is an inference across experiments rather than an observation. Against the first standard the sixteen targets account as follows. Three—GLP-1R, PDE4 and CB1—are supported on both sides with both organs moving in the desired direction, each of them under one strategy applied in separate experiments rather than in one system. Three move the two sides in opposite directions: FGF21 through its muscle arm, mGluR5 through a sign that reverses with the liver cell engaged, and GRP–GRPR through two sides that would need opposite pharmacology. The remaining ten have had no manipulation read out on both organs, and they divide evenly. Five have one side not evaluated in an alcohol context: no hepatic amylin-receptor action in any alcohol model, no alcohol-indication liver evidence for the oxytocin receptor, and no peer-reviewed brain-side manipulation of bile-acid receptors, cytolysin or IL-22–REG3G. Five are supported on both sides, but their two sides come from different manipulations—or, at GDF15–GFRAL, from association on the drinking side—so no one strategy has been read out on both organs: GIPR, GHSR–LEAP2, GDF15–GFRAL, TLR4–HMGB1–RAGE and IL-1β–NLRP3–IL-1R1. Three plus three plus ten is sixteen, and five plus five is ten (Table S1). What looks dual is often two literatures sharing a molecule’s name, not one molecule tested twice.

The asymmetry runs the other way too. Three targets carry a negative human trial on the brain side, and none carries an adequately powered placebo-controlled negative trial on the liver side, the hepatic failures turning on power or on comparator design rather than on the target. That is why they motivate a dual-endpoint design rather than retire a target.

Much of this lies in experimental systems. Almost every liver-side mechanistic claim here arose in the chronic-plus-binge mouse model, where ethanol is administered and not chosen, so no drinking endpoint can exist in it [137]. Conversely, free-choice drinking in selectively bred mice held blood ethanol above 80 mg/dL and induced CYP2E1, yet altered neither hepatic histology nor alcohol-metabolising enzymes [138]. The two sides of most targets thus come from non-overlapping systems—contingently so, since outside these target families loss of ketohexokinase-A/C in mice reduced alcohol preference across three paradigms and protected the liver under pair-matched ethanol, in the same models [139]. Timing is the second systematic error: the hepatic interleukin-1 agents reached patients with established severe disease while the mouse work dosed prophylactically [9,126].

Three limits belong to this way of reading the evidence. Agreement between the two sides is no formality. Hepatic mGluR5 engagement is injurious on Kupffer cells and protective on natural killer cells [15,99]. Bombesin-receptor activation lowers drinking—with a ligand that is not GRPR-selective—where hepatic benefit requires blockade [105,106]. In neither case can a single systemic ligand have the right sign in both compartments. Organ-selective pharmacology is the escape, demonstrated preclinically at one receptor only: peripheral restriction delivers both arms at CB1 [18,104], though no peripherally restricted CB1 antagonist has reached clinical stage and none for mGluR5 has been reported at all. The axis also runs backwards: female mice with ethanol-diet-induced early steatosis then drank more, intake correlating with steatosis severity [25], so a hepatoprotective drug may reduce drinking because it protects the liver. That steatosis was transient and ethanol-induced, however, leaving prior exposure an alternative explanation, and the mechanism was inferred from BDNF/TrkB rather than tested by manipulation [25]. Appraising the evidence organ by organ has its own blind spots: it cannot weigh bidirectionality, a community phenotype that is not a molecule, or unverified target exposure, as with intranasal oxytocin [134].

Endpoints decide what a trial can show, and the criterion for one decides how many count as dual (Section 2.3). A validated biomarker is not histology [43,44], and only one retrieved trial carried a histological primary endpoint (Table 1). The ceiling on human evidence matters as much. Across eight emulated target trials incident substance use disorders fell for six substance classes, and that uniformity, not the hazard ratio, is the critical reading [140]. Pooled, alcohol intake was unchanged while alcohol-related and liver-related events fell [141], matching the null randomised summary for consumption and craving [58]. Both syntheses closed their searches in May 2025, before the 2026 randomised reports, which sharpens rather than weakens the point that pharmaco-epidemiology, which ranks below a randomised trial throughout this review, cannot close that gap. Combination logic inherits every limit: the two combinations tested in people, both in MASH, missed the fibrosis endpoint where one single agent did not [67], and FGF19 with ethanol worsened hepatic inflammation in mice [117].

Hepatic and nutritional safety in alcohol-related cirrhosis, finally, is not yet shown. Semaglutide did not improve fibrosis in MASH-related compensated cirrhosis, with no decompensating events or deaths [142]. Survodutide needed no dose adjustment across Child–Pugh classes, including decompensated cirrhosis [143], and cagrilintide exposure was unchanged in hepatic impairment [66]. These are phase 1 and MASH data, none of it in an alcohol population, where the one cohort with a hepatic progression endpoint was null [75]. The single registered trial in ALD excludes prior decompensation, liver stiffness ≥25 kPa, grade ≥2 varices and lean patients, its body-mass-index floor being above 25 kg/m^2^, so it cannot settle it either (NCT06409130, checked 17 October 2026). Selection follows measurement (Section 2.3) [4,41]: strata belong on measured intake, not self-report.

## 8. Future Directions

In a 2018 appraisal of trial design the field named two of its own gaps—no plausible animal model, no consensus composite endpoint—and both are still open [144]. What follows is written as experiments rather than priorities.

The hepatic arm of each dual target should be deleted conditionally, in the cell type the mechanism names and in descending order of how well the target is otherwise supported: endothelial *Glp1r*, myeloid *Oxtr*, hepatocyte *Slc17a8*, each under chronic-plus-binge ethanol and read out on histology and, where the paradigm allows, on intake—on the precedent of the hepatocyte-specific FGF21 knockout [79]. The model that would make the second of those readouts routine does not exist: one coupling dependence-like voluntary intake with advanced fibrosis, which means intermittent-access drinking over months in a high-drinking line, with pair-matched forced-feeding controls [137,138] and fibrosis stage, not steatosis, as the primary readout [139]. Genetic triangulation belongs before phase 2 rather than after it, as a prospective filter: two independent instruments with colocalisation and both a drinking and a liver outcome, as achieved for FGF21–*KLB* [82] and required first of GDF15–GFRAL and GRPR.

In the clinic, every dual-target trial needs co-primary PEth-verified intake and a liver endpoint [38], with disclosure of the fibrosis result prespecified, since fibrosis screening with counselling was followed by 45% abstinence at six months [46]. At one target at a time, peripherally restricted and brain-penetrant agents should be compared head-to-head with intake and histology measured in the same animals: feasible today at CB1 [18,104], awaiting a tool compound at mGluR5. Any microbial carriage-to-outcome association needs prespecified multicentre replication with strain-level typing before a further phage or transplantation programme [111]. And the population the one registered ALD trial excludes needs a study of its own: a prospective safety and body-composition study of an incretin in Child–Pugh A–B alcohol-related cirrhosis, with decompensation, muscle mass and PEth-verified intake as endpoints (NCT06409130, checked 17 October 2026).

Two further gaps are obligations rather than experiments. Completed trials have to be reported: for GLP-1 receptor agonists in AUD alone, 19 interventional studies are in progress or completed-but-unpublished against four published [145], and separately an FGF21 infusion with an alcohol-preference endpoint, a GET 73 bar-laboratory and spectroscopy study and the combination trial in ALD have all completed without reported results (NCT04232033, NCT04831684, NCT06409130; checked 17 October 2026) (Table 1). And the one-sided targets have to be closed: human genetics for GDF15–GFRAL, a GRPR ligand tested on intake and histology in the same animals, a brain-side bile-acid receptor manipulation with a drinking endpoint, and a cell-type-conditional brain IL-1R1 deletion, since the two existing manipulations disagree [129].

## 9. Conclusions

Alcohol-related liver injury and the drinking that drives it are governed in part by the same receptors, ligands and transporters along the gut–liver–brain axis, so abstinence can be addressed as a molecular target and not only a behaviour. Being supported on both sides and being demonstrated to act on both organs in the same experimental system are different tests; the first is commoner than the second, and no target of the sixteen meets the second at all. All four target families carry evidence on both sides: incretin and appetite-regulating receptors, hepatokines, glutamatergic signalling, and the microbiome-derived and shared neuroimmune layer. Of the sixteen targets, three are supported on both sides with both organs also moving in the desired direction, each under one strategy applied in separate experiments rather than in one system: GLP-1R, PDE4 and CB1, two of them in the neuroimmune layer whose evidence Section 6.5 holds below its mechanistic detail. Three point both ways at once—FGF21 through its muscle arm, mGluR5 through a sign that reverses with the liver cell engaged, and GRP–GRPR through two sides that would need opposite pharmacology. The remaining ten have had no manipulation read out on both organs: five because one side has not been evaluated in an alcohol context, and five because their two sides rest on different manipulations. A molecule’s name is shared more often than a molecule is tested twice. Priority should follow the strength of the evidence rather than attention: human genetic triangulation backed by a preclinical mechanism that points the same way ranks highest, though no target yet combines it with both organs shown to move in the desired direction, pharmaco-epidemiology lowest, and the failed, terminated and non-replicated results calibrate the field as surely as the positive ones. For trial design it follows that a liver endpoint and an objectively measured intake endpoint have to be co-primary: a biomarker endpoint and a histological endpoint are not interchangeable, and a dual mechanism measured on one organ will keep being read as a single-organ effect. Hepatic safety in established alcohol-related cirrhosis remains unresolved rather than reassuring.

## Figures and Tables

**Table 1:** Human studies that measure a liver outcome and an alcohol-use outcome, and the single-endpoint programmes needed to read them. All design, population, arm, endpoint, enrolment, status and date fields were read from the ClinicalTrials.gov record on 17 October 2026; a registry record supports design and status only and never a result. Result cells are therefore either carried by a cited publication, by numbers the sponsor has posted in the registry (labelled as such), or read “results not published”. Of 64 verified registry records, one makes a liver outcome and an alcohol-use outcome both primary (NCT07060638), and six more pair the two with exactly one side primary: the liver endpoint primary in two (NCT06409130, NCT07046819) and a consumption, abstinence or intake-biomarker endpoint primary in four (NCT05895643, NCT06546384, NCT06939088, and NCT06496191, whose registry status is no longer verified). Two further records measure both sides but qualify on neither count—one keeps both sides secondary behind a cue-elicited-craving primary (NCT06727331), and one carries liver enzymes as secondary endpoints with no consumption endpoint at all (NCT07148843)—because an alcohol-use endpoint is counted here as a consumption, abstinence or intake-biomarker measure, not as craving, cue reactivity or withdrawal severity. No retrieved trial uses histology together with an objectively measured intake endpoint as co-primaries. ClinicalTrials.gov identifiers are given inline and are not reference-list entries.

NCT Number	Agent(s) and Target(s)	Population	Phase, n, Status (Verified)	Liver Endpoint	Alcohol-Use Endpoint	Dual by	Result or Status (Checked 17 October 2026)	Refs.
NCT06409130	Zalfermin (NNC0194-0499, reported as an FGF21 analogue) + cagrilintide + semaglutide; 7 arms	Alcohol-related liver disease	Ph 2, n = 270 actual, completed (March 2026)	ELF score (primary); Pro-C3, VCTE, CAP, ALT, AST (secondary)	PEth and TLFB (both secondary)	Design, liver-primary	Start 20 May 2024, primary completion 21 November 2025, study completion 12 January 2026; no results posted and no publication indexed. The liver primary is a biomarker, not histology, and the liver endpoint is attributable only to the combination	results not published
NCT07060638 (ITAALD)	IL-22 (F-652) + acamprosate vs. prednisone + motivational therapy	Alcohol–associated hepatitis	Ph 2, n = 216 est., recruiting (July 2026)	Death, transplant, ascites, hepatic encephalopathy, variceal bleeding, liver-related admission, MELD rise >5 (all primary, 6 months)	Return to drinking (primary, 6 months)	Design, co-primary	Start 27 January 2026; estimated primary completion December 2029. Dual by combination, not by one molecule	pending
NCT07046819	Tirzepatide (GIPR/GLP-1R)	MetALD with AUD	Ph 2, n = 120 est., recruiting (July 2026)	CAP steatosis with body weight (single composite primary); MRI spectroscopy, visceral fat, ALT/AST/GGT (secondary)	Drinking behaviours or cravings and WHO risk drinking level, inside one composite secondary	Design, liver-primary; drinking inside one composite secondary	Start 11 June 2026; estimated primary completion 30 July 2027. Not co-primary	pending
NCT06546384	Semaglutide (GLP-1R) with weight-reduction advice, open label	Obesity with fatty liver and AUD	Ph NA, n = 64 est., not yet recruiting (February 2026)	FibroScan elastography and FIB-4 (secondary)	Total abstinence by negative PEth (primary); TLFB, urinary EtG, heavy drinking days (secondary)	Design, alcohol-primary	Estimated start 1 November 2026; the only retrieved record whose primary endpoint is PEth-defined abstinence	pending
NCT05895643	Semaglutide (GLP-1R) with cognitive behavioural therapy	AUD with comorbid obesity	Ph 2, n = 108 actual, completed (August 2025)	FIB-4, GGT, ALAT (prespecified secondary)	Heavy drinking days (primary); PEth, WHO risk levels, PACS, AUDIT (secondary)	Design, alcohol-primary	Heavy drinking days −13.7 percentage points (95% CI −22.0 to −5.4; *p* = 0.0015) at 26 weeks. No results posted in the registry and no liver-enzyme value is available in the published abstract, so none is given here	[34]
NCT03232112	Exenatide (GLP-1R)	Alcohol dependence with obesity	Ph 2, n = 152 actual, completed (June 2021)	None (SF-36 only)	Heavy drinking days (primary); PEth, PACS, AUDIT (secondary)	Alcohol only; PEth secondary analysis	No overall reduction in heavy drinking days, though cue reactivity and an obesity-restricted subgroup changed; in the secondary analysis PEth fell by 0.9 µmol/L vs. placebo, and only at week 26	[35,36]
NCT06727331	Tirzepatide (GIPR/GLP-1R)	AUD	Ph 2, n = 20 est., recruiting (July 2026)	FIB-4 (1 of 18 secondary endpoints)	Cue-induced craving (primary, with adverse events); percentage heavy drinking days, drinks per drinking day	Design; both sides present, both secondary (craving primary)	Recruiting; no results	pending
NCT06939088	Tirzepatide (GIPR/GLP-1R)	AUD with schizophrenia	Ph 2, n = 108 est., recruiting (February 2026)	Liver fibrosis by FIB-4 (1 of 28 secondary endpoints)	Heavy drinking days (primary, 16 weeks)	Design, alcohol-primary	Recruiting; no results	pending
NCT06015893 (STAR)	Semaglutide (GLP-1R) with a behavioural platform	AUD	Ph 2, n = 63 actual, active, not recruiting (31 August 2026)	None	Drinks per week (primary); functional MRI, virtual-reality buffet, bar-laboratory craving, PEth (secondary)	Alcohol only	Active, not recruiting; estimated primary completion 31 March 2027, so not completed	pending
NCT06136195	Mavoglurant (mGluR5 negative allosteric modulator), crossover	Heavy drinkers and AUD	Ph 1, n = 63 est., recruiting (June 2026)	None	Drinks consumed and craving (both primary, two laboratory sessions)	Alcohol only	Start 1 July 2024; estimated primary completion 31 May 2028. Recruiting, not completed, and no human alcohol efficacy result exists for mavoglurant	pending
NCT04232033	Recombinant FGF21 infusion vs. saline	Healthy men	Ph NA, n = 20 actual, completed (December 2024)	Plasma FGF21 (secondary)	Alcohol preference on a visual analogue scale (primary)	Alcohol only (brain side)	Primary completion 14 June 2022; no results posted and no publication linked. The only registered human experiment that manipulates FGF21 and measures an alcohol-related outcome	completed, unreported
NCT04563026 (AHFIRM)	Larsucosterol (DUR-928) 30 or 90 mg, DNA methyltransferase inhibitor	Alcohol–associated hepatitis	Ph 2b, n = 307 actual, completed (September 2024)	90-day mortality or liver transplant (primary)	None	Liver only (comparator)	Primary endpoint not met; no results posted in the registry, so the result is taken from the publication	[8]
NCT04072822	Anakinra (IL-1 receptor antagonist) + zinc vs. prednisone	Alcohol–associated hepatitis	Ph 2, n = 147 actual, completed (February 2025)	Survival at 90 days (primary)	None (gut-permeability indicators among secondary endpoints)	Liver only (comparator)	Registry-posted 90-day survival 90% with prednisone vs. 70% with anakinra plus zinc	[9]
NCT03775109 (ISAIAH)	Canakinumab (IL-1beta)	Alcohol–associated hepatitis	Ph 2, n = 55 actual, completed (February 2025); the publication reports 57 randomised	Histological improvement on day-28 biopsy (primary): the only histological primary endpoint in the retrieved set	None	Liver only (comparator)	Registry-posted 14/28 vs. 10/27 improved; *p* = 0.248, net mean difference 16.7 (95% CI −11.2 to 44.5)	[10]
NCT05639543 (FRESH)	INT-787 (FXR agonist)	Severe alcohol–associated hepatitis	Ph 2, n = 67 actual, terminated (April 2026)	Lille model response on day 7 (primary)	None	Liver only (comparator)	Terminated 31 March 2026; the registry gives a business decision taken because there was no clear evidence of potential benefit in severe AH, and states it was not a safety concern. Nothing posted or published	registry record only
NCT06750588	NTR-101 oral bacteriophage preparation (cytolytic *Enterococcus faecalis*)	Alcohol–associated hepatitis	Ph 1, n = 12 est., not yet recruiting (August 2025)	Adverse-event frequency and change in liver function (primary)	None	Liver only (comparator)	The estimated start of 5 November 2025 has passed with no registry update, so the record is stale; registry-linked preclinical rationale only	[37]

Status wording is the registry’s own. “Active, not recruiting” and “not yet recruiting” are not “completed”; where a record’s estimated start or completion date has passed without a registry update, the staleness is stated rather than resolved. Registry-posted results are the sponsor’s own posted numbers and are labelled; they are not a publication. “Results not published” means no results are posted in the registry and no publication is indexed against the identifier in PubMed, checked 17 October 2026. Abbreviations (Table 1). ALAT, alanine aminotransferase (registry spelling); ALD, alcohol–associated liver disease; ALT, alanine aminotransferase; AH, alcohol–associated hepatitis; AST, aspartate aminotransferase; AUD, alcohol use disorder; AUDIT, Alcohol Use Disorders Identification Test; CAP, controlled attenuation parameter; CI, confidence interval; ELF, Enhanced Liver Fibrosis score; EtG, ethyl glucuronide; FGF21, fibroblast growth factor 21; FIB-4, fibrosis-4 index; FXR, farnesoid X receptor; GGT, γ-glutamyl transferase; GIPR, glucose-dependent insulinotropic polypeptide receptor; GLP-1R, glucagon-like peptide-1 receptor; IL, interleukin; MASH, metabolic dysfunction–associated steatohepatitis; MELD, Model for End-stage Liver Disease; MetALD, metabolic dysfunction–associated alcohol-related liver disease; mGluR5, metabotropic glutamate receptor 5; MRI, magnetic resonance imaging; PACS, Penn Alcohol Craving Scale; PEth, phosphatidylethanol; ph, phase; Pro-C3, N-terminal pro-peptide of type III collagen; SF-36, 36-Item Short Form Health Survey; TLFB, timeline followback; VCTE, vibration-controlled transient elastography; WHO, World Health Organization. *Enterococcus faecalis* is italic.

## Data Availability

No new experimental data were created. All evidence reviewed here is from published articles, cited in the reference list. The trial census underlying Table 1 was assembled from the ClinicalTrials.gov registry and verified against it on 17 October 2026; the caption of Table 1 states the eligibility criterion, the read date, the number of records screened and the identifier of every record that qualified. The full census, including the records screened and excluded, is available on request from the corresponding author.

## Supplementary Materials

The following supporting information can be downloaded at: https://www.mdpi.com/article/10.3390/ijms27219921/s1.

## Abbreviations

The following abbreviations are used in this manuscript:

AH	alcohol–associated hepatitis
ALD	alcohol–associated liver disease
AMP	adenosine monophosphate
ADRB2	β2-adrenergic receptor (gene *ADRB2*)
AMY1R	amylin receptor 1
ASC	apoptosis-associated speck-like protein containing a CARD
AUD	alcohol use disorder
BDNF	brain-derived neurotrophic factor
BLA	basolateral amygdala
CB1	cannabinoid receptor type 1
CD4	cluster of differentiation 4
CD8	cluster of differentiation 8
CeA	central nucleus of the amygdala
ChREBP	carbohydrate-response-element-binding protein
CI	confidence interval
CNS	central nervous system
CoA	coenzyme A (acetyl-CoA, acetyl coenzyme A)
COVID-19	coronavirus disease 2019
CREB	cyclic-AMP-response-element-binding protein
CXCL1	C-X-C motif chemokine ligand 1
CYP2E1	cytochrome P450 2E1
EAAT2	excitatory amino-acid transporter 2
ELF	Enhanced Liver Fibrosis (score)
FGF15, FGF19, FGF21	fibroblast growth factor 15, 19 and 21
FGFR1, FGFR3	fibroblast growth factor receptor 1 and 3
G3P	glycerol-3-phosphate (not glyceraldehyde-3-phosphate)
FXR	farnesoid X receptor
G-CSF	granulocyte colony-stimulating factor
GABA	γ-aminobutyric acid (GABA-A, its type A receptor)
GDF15	growth differentiation factor 15
GDNF	glial cell line-derived neurotrophic factor
GFRAL	GDNF-family receptor α-like
GOAT	ghrelin O-acyltransferase (gene *MBOAT4*)
GHSR	growth hormone secretagogue receptor
GIP	glucose-dependent insulinotropic polypeptide
GIPR	GIP receptor (gene *GIPR*)
GLP-1	glucagon-like peptide-1
GLP-1R	GLP-1 receptor (gene *GLP1R*, mouse *Glp1r*)
GRP	gastrin-releasing peptide
GRPR	GRP receptor (gene *GRPR*, mouse *Grpr*)
HMGB1	high-mobility group box 1
IL	interleukin
IL-1R1	interleukin-1 receptor type 1 (gene *Il1r1*)
IL-1R2	interleukin-1 receptor type 2
IL-1Ra	interleukin-1 receptor antagonist
IRF1	interferon regulatory factor 1
KLB	β-Klotho (gene *KLB*, mouse *Klb*)
LEAP2	liver-expressed antimicrobial peptide 2
LSEC	liver sinusoidal endothelial cell
Ly6C	lymphocyte antigen 6 complex locus C
MASH	metabolic dysfunction–associated steatohepatitis
MASLD	metabolic dysfunction–associated steatotic liver disease
MetALD	metabolic dysfunction–associated alcohol-related liver disease
mGluR, mGluR5	metabotropic glutamate receptor, and its subtype 5 (gene *GRM5*, mouse *Grm5*)
NADPH	nicotinamide adenine dinucleotide phosphate
NCT	ClinicalTrials.gov registry identifier
NIAAA	National Institute on Alcohol Abuse and Alcoholism
NK	natural killer (cell)
NLRP3	NOD-, LRR- and pyrin-domain-containing protein 3 (gene *Nlrp3*)
NOX2	NADPH oxidase 2 (gene *Cybb*)
NR4A1	nuclear receptor subfamily 4 group A member 1
NRF2 (*Nfe2l2*)	nuclear factor erythroid 2-related factor 2, the antioxidant master regulator
OXTR	oxytocin receptor (gene *Oxtr*)
PDE, PDE4	phosphodiesterase, phosphodiesterase 4
PCSK9	proprotein convertase subtilisin/kexin type 9
PET	positron-emission tomography
PEth	phosphatidylethanol
PI3K	phosphoinositide 3-kinase
PKC	protein kinase C
PTEN	phosphatase and tensin homolog
RAGE	receptor for advanced glycation end-products
RAMP1	receptor activity-modifying protein 1
REG3G	regenerating islet-derived protein 3γ (gene *Reg3g*)
RNA	ribonucleic acid
SIRT1	sirtuin 1
SLC7A11	solute carrier family 7 member 11, the gene encoding xCT
SNARE	soluble *N*-ethylmaleimide-sensitive-factor attachment protein receptor
TGR5	Takeda G-protein-coupled receptor 5 (gene *GPBAR1*)
TLR, TLR4	Toll-like receptor, Toll-like receptor 4 (gene *Tlr4*)
TNF-α	tumour necrosis factor α
TrkB	tropomyosin receptor kinase B
US	United States
VGLUT3	vesicular glutamate transporter 3 (gene *SLC17A8*, mouse *Slc17a8*)
xCT	light-chain subunit of the cystine–glutamate antiporter system xc
ZO-1	zonula occludens-1
