# Supplementary material for: Dual Targeting of Alcohol Use Disorder and Alcohol–Associated Liver Disease: Emerging Molecular Targets Along the Gut–Liver–Brain Axis
Source: Int J Mol Sci. 2026 Nov 5;27(21):9921. doi: 10.3390/ijms27219921 (PMC13764431; doi:10.3390/ijms27219921)
Supplement: Supplementary file 1 [file ijms-27-09921-s001.zip › ijms-4677892-supplementary.pdf]

Supplementary Materials

Table S1. Candidate molecular targets of the gut–liver–brain axis, with the evidence on the drinking side and on the hepatic side set out side by side. For each target the table gives the cell types and the experimental evidence on each side, the human genetic or biomarker evidence where any exists, the clinical-stage agent and its trial identifier where one exists, and the cited sources. Negative, terminated and non-replicated results are shown with the same prominence as positive ones. ClinicalTrials.gov identifiers are given inline and are not reference-list entries. This table supports Sections 3–6 of the main text. The column "How the two sides relate" states, for each target, whether the drinking-side and the liver-side evidence come from the same experimental system, from different systems, or whether one side has not been evaluated at all, and flags a side that rests on association alone. **No target in the census has had both organs read out in the same experimental system.** The sixteen targets account as follows: three move both organs in the desired direction, although in separate experiments under the same strategy (GLP-1R, PDE4, CB1); three move them in opposite directions (FGF21, mGluR5, GRP–GRPR); and ten have had no manipulation read out on both. Those ten divide into five with one side not evaluated in an alcohol context (calcitonin–amylin receptor, oxytocin receptor, FXR/TGR5, cytolysin, IL-22–REG3G) and five supported on both sides but never by a single manipulation (GIPR, GHSR–LEAP2, GDF15–GFRAL, TLR4–HMGB1–RAGE, IL-1β–NLRP3–IL-1R1). Three plus three plus ten is sixteen.

**Target (protein; gene) and family**	**Cell type(s) and brain/drinking evidence**	**Cell type(s) and liver evidence**	**How the two sides relate**	**Human genetics or biomarker**	**Agent and phase (NCT)**	**Key refs**
GLP-1R; *GLP1R*. Incretin/appetite (Section 3)	Cells: dLS GLP-1R neurons; hindbrain NTS. Liraglutide requires dLS *Glp1r* (M); septal agonism lowers intake with food and water unchanged (M, R), though the effect is not alcohol-selective across two species and both sexes (M, R); semaglutide -13.7 pp heavy drinking days (H); exenatide null (H, neg)	Cells: pericentral LSEC, CD8+ T cells, not hepatocytes§. Exendin-4 lowers ALT and hepatic TG in established ALD (R); semaglutide suppresses lipogenesis (M); endothelial *Glp1r* loss abolishes benefit (M)§	Different experimental systems — the same strategy moves each organ, but in separate experiments; never both in one system	Drug-target MR separates *GLP1R* from *GIPR* (HG); PEth-confirmed intake reduction at week 26 (H); T2 contested: one target-trial emulation positive for a liver composite and an alcohol-use outcome in the same cohort; a larger active-comparator cohort in an alcohol population null	Semaglutide ph 3 AUD (NCT07218354), ph 2 AUD (NCT05895643, NCT06015893), ph 2 ALD arm (NCT06409130)	
GIPR (GIP/GLP-1 co-agonism); *GIPR*. Incretin/appetite (Section 3)	Cells: lateral septum; mesolimbic reward. Tirzepatide attenuates reward and cuts intake, binge and relapse-like drinking without tolerance (R, M)	Cells: not resolved. Hepatic triglyceride falls in the same alcohol-exposed animals, but in a metabolic rather than an alcohol-injury context; no histology and no conditional knockout (R)	Different experimental systems — both sides supported, but no single manipulation has been read out on both; liver side is association only	Lower incident AUD with tirzepatide and semaglutide, not liraglutide or dulaglutide (H, T2); independent *GIPR* MR locus (HG)	Tirzepatide ph 2 AUD (NCT06727331, NCT06939088), ph 2 MetALD (NCT07046819)	
Calcitonin–amylin receptor (AMY1R, CTR); *CALCR*. Incretin/appetite (Section 3)	Cells: accumbens calcitonin receptor, RAMP1. Salmon calcitonin lowers intake, relapse-like drinking and self-administration but not chocolate, and AC187	Cells: none shown. Not shown: no hepatic amylin-receptor action in any alcohol model	One side not evaluated — no liver-side evidence in an alcohol context	None; cagrilintide exposure unchanged in mild-to-severe hepatic impairment (H, pharmacokinetic safety, not efficacy)	Cagrilintide ph 2 ALD combination arm (NCT06409130, completed, unreported)	

	raises intake (R); a selective agonist loses effect, with a sex difference (R, null)					
GHSR with LEAP2; *GHSR*, *LEAP2*. Incretin/appetite (Section 3)	Cells: amygdala; mesolimbic dopamine; nodose afferents. Intravenous ghrelin raises self-administered alcohol 25% and the amygdala cue signal, so agonism worsens (H); the inverse agonist did not reduce cue-elicited craving while its food effect survived, in a trial terminated for COVID-19 (H, uninformative); central LEAP2 lowers intake (M)	Cells: not hepatocyte-autonomous (pancreas–adipose–liver axis). GHSR blockade suppresses adipose lipolysis and attenuates alcohol-related steatosis (R); GHSR-knockout rats resist ALD (R)	Different experimental systems — both sides supported, but no single manipulation has been read out on both	None: serum LEAP2 does not track consumption, and no human ghrelin study carries a liver endpoint	PF-05190457 ph 2 AUD terminated for COVID-19, T1(term) (NCT02707055); GLWL-01 ph 1/2 AUD completed (NCT03896516)	
FGF21 with beta-Klotho; *FGF21*, *KLB*. Hepatokine (Section 4)	Cells: KLB+ basolateral amygdala neurons. *KLB* associated with consumption plus brain-specific beta-Klotho knockouts drinking more, in one paper (HG, M); an analogue cuts intake 50% (NHP); FGF21-knockout rats drink normally (R, null)	Cells: hepatocytes (ChREBP, SIRT1); muscle satellite cells (harm arm, in decompensated cirrhosis of unspecified aetiology, not an alcohol model)‡. hepatocyte-specific FGF21 loss worsens acute-on-chronic injury (M); but *Fgf21*-knockouts are protected via adipose lipolysis (M, opposite), while in FGF21-knockout rats on a Gubra-Amylin-NASH diet plus alcohol its loss mildly worsened transaminases and hepatic triglycerides, with no histological change (R)	Different experimental systems — and the two sides move in opposite directions	MR: higher FGF21 with less problematic alcohol use and lower ALD risk, replicated with a second instrument (HG); serum FGF21 highest in moderate-to-severe AH (H, opposite)	Efruxifermin reached ph 2b MASH with positive histology§, yet zalfermin + semaglutide missed the 52-week MASH fibrosis endpoint, T1(neg)§; ph 2 ALD arm (NCT06409130)††; FGF21 infusion unreported (NCT04232033)	
mGluR5 with VGLUT3; *GRM5*, *SLC17A8*. Glutamatergic (Section 5)	Cells: limbic and temporal neurons (imaging only). Availability is lower in limbic regions in one study but higher in temporal regions in another, so the human	Cells: mGluR5 sits on three recipient cells with opposite consequences - Kupffer, stellate and NK. Kupffer: binge-triggered Ca2+/SNARE-dependent hepatocyte release activates mGluR5-PKC-	Different experimental systems — and the two sides move in opposite directions; drinking side is association only	Epigenome-wide lead probe in *SLC7A11* (H); plasma glutamate correlates with ALT, AST and GGT (H, n = 16, with no elevation in cirrhosis)	Mavoglurant ph 1 in heavy drinkers (NCT06136195; its only efficacy result is in cocaine use disorder); GET 73 ph 1/2 AUD completed, unreported (NCT04831684); no	

	direction is contradictory (H); a compound with putative mGluR5 activity did not change laboratory craving or self-administration (H, neg)	NOX2, and Clec4f-cre *Grm5* or *Cybb* deletion protects (M, HT). Stellate: alcohol induces mGluR5, driving 2-AG onto hepatocyte CB1 and lipogenesis (global and pharmacological inhibition, not stellate-conditional) (M). NK: mGluR5 is anti-fibrotic, so its loss aggravates fibrosis, in carbon tetrachloride and methionine-choline-deficient models (M)‡			peripherally restricted ligand reported	
GDF15 with GFRAL; *GDF15*, *GFRAL*. Hepatokine (Section 4)	Cells: area postrema and NTS GFRAL neurons. *Gfral*-knockouts resist stress and chemotherapy anorexia, but no study carries an alcohol readout (M)‡; GDF15 anorexia works through nausea (M)‡	Cells: perivenous hepatocytes (ADRB2-CYP2E1); Kupffer cells; senescent hepatocytes. Alcohol induces GDF15 in primary hepatocytes and *Gdf15*-knockouts have worse injury (M); gut catecholamines on hepatocyte ADRB2 raise GDF15, which kills inflammatory Kupffer cells (M, HT)	Different experimental systems — both sides supported, but no single manipulation has been read out on both; drinking side is association only	Plasma GDF15 predicts corticosteroid response and 90-day mortality in AH in two cohorts (H, prognostic association); no peer-reviewed human genetics in alcohol consumption	None with an alcohol or liver indication; recombinant GDF15 is preclinical, with weight-loss independence shown in MASH§	
TLR4 with the HMGB1–RAGE module; *TLR4*, *HMGB1*, *AGER*. Neuroimmune (Section 6)	Cells: central-amygdala neurons; hippocampus (HT). Central-amygdala but not ventral-pallidal TLR4 knockdown reduces binge drinking, with a sucrose control (R); HMGB1-IL-1beta complexes are raised in post-mortem alcoholic hippocampus and induced by binge ethanol (HT, M)	Cells: hepatocytes (TLR4, not myeloid); Kupffer cells, macrophages (RAGE). Hepatocyte-specific, not myeloid-specific, *Tlr4* deletion protects against alcohol-induced fatty liver (steatosis endpoints; no fibrosis or hepatitis model) (M); hepatocyte oxidised HMGB1 complexed with IL-1beta signals through myeloid RAGE, is present in human AH liver and travels to the gut (M, HT)	Different experimental systems — both sides supported, but no single manipulation has been read out on both	No human genetic evidence; human tissue only	TAK-242 with or without G-CSF, ph 2 in severe AH and acute-on-chronic liver failure (NCT06890039)	
IL-1beta with NLRP3 and IL-1R1; *IL1B*, *NLRP3*, *IL1R1*. Neuroimmune	Cells: basolateral (not central) amygdala; corticostriatal; cell type	Cells: Kupffer cells (caspase-1/ASC). IL-1R1 with caspase-1/ASC	Different experimental systems — both sides supported, but no single	None	Anakinra ph 2 AH (NCT04072822) and canakinumab ph 2 AH	

(Section 6)	unresolved. IL-1 receptor antagonist in the basolateral but not central amygdala lowers binge drinking (sucrose and locomotor controls, M); *Nlrp3* deletion lowers binge intake (M), though neither prevented withdrawal-induced escalation of two-bottle-choice drinking; and constitutive *Il1r1* deletion leaves intake and preference unchanged (M, null)	maturation is required for alcohol-induced injury and IL-1 receptor antagonist reverses it (M); interventional evidence uninformative: canakinumab underpowered (histological endpoint, nominally positive post hoc); anakinra inferior to prednisone in an active-comparator design with zinc co-administered - neither tests IL-1 blockade against placebo	manipulation has been read out on both		(NCT03775109), both completed; neither informative, see the liver evidence cell	
PDE4 (PDE4B); *PDE4B*. Neuroimmune (Section 6)	Cells: nucleus accumbens neurons. Apremilast lowers binge-like and dependence-related drinking across mouse backgrounds, and reduced drinking in a phase IIa study (M, H); ibudilast changed no drinking or inflammatory outcome in the powered trial (H, neg)	Cells: not resolved. The PDE4 inhibitor ZL40 reduced alcohol intake and alleviated alcohol-related liver injury in one compound series (M)	Different experimental systems — the same strategy moves each organ, but in separate experiments; never both in one system	None	Apremilast (approved elsewhere) ph 2 AUD (NCT07029529, NCT07325266); ibudilast ph 2 AUD, T1(neg) (NCT03594435)	
CB1; *CNR1*. Neuroimmune (Section 6)	Cells: Gpr65+ nodose-ganglion vagal afferents, not brain neurons. The anti-drinking effect of non-brain-penetrant antagonists requires CB1 on Gpr65+ nodose vagal afferents and is interdependent with ghrelin-receptor signalling in the same neurons (M)	Cells: hepatocytes (CB1); stellate cells (2-AG source). Ethanol induces stellate-cell 2-arachidonoylglycerol acting on hepatocyte CB1, and hepatocyte-specific knockouts are protected (M); a peripherally restricted antagonist is efficacious against alcohol-related steatosis (M)	Different experimental systems — the same strategy moves each organ, but in separate experiments; never both in one system	None	No clinical-stage peripherally restricted CB1 antagonist; the registered AUD cannabidiol trials are not selective CB1 antagonists	
Oxytocin receptor; *OXTR*. Neuroimmune (Section 6)	Cells: not resolved (intranasal; central exposure uncertain). In the largest randomised multisite trial intranasal oxytocin had no effect on heavy drinking days, craving or mood (H, neg)	Cells: myeloid-derived hepatic macrophages, not stellate cells‡. Oxytocin reverses fibrosis through their receptors via Ca2+/CREB/NR4A1 and a Ly6C switch, in carbon tetrachloride and bile-	One side not evaluated — no liver-side evidence in an alcohol context	None of citable quality	Intranasal oxytocin ph 2 to 2/3 AUD; all three records completed (NCT03878316, NCT04523922, NCT05093296)	

		duct-ligation models only (M)‡				
FXR and TGR5 with FGF15/FGF19; *NR1H4*, *GPBAR1*, *FGF19*. Microbial effector (Section 6)	Cells: none shown. Not shown: no peer-reviewed brain-side bile-acid study with an alcohol endpoint exists	Cells: enterocytes (FXR, FGF15); hepatic macrophages (TGR5); hepatocytes. Ethanol-enriched bile-salt hydrolase lowers enterocyte FXR and FGF15; fexaramine and FGF19 protect (M); global FXR loss is worse while hepatocyte-specific loss is null; TGR5 loss worsens injury via macrophage recruitment (M, female mice only); but AAV-FGF19 overexpression plus ethanol increased inflammation (M, female, 10-day chronic-plus-binge, harm)	One side not evaluated — no drinking-side evidence in an alcohol context	Serum bile acids and FGF19 markedly raised in AH versus decompensated cirrhosis and health, in two cohorts (H, associative)	INT-787 ph 2 severe AH terminated 2026-03-31 for no clear evidence of benefit, not safety, T1(term) (NCT05639543); obeticholic acid mechanistic study in heavy drinkers (NCT02654236)	
Cytolysin of *Enterococcus faecalis* (phage-targetable). Microbial effector (Section 6)	Cells: none shown. Not shown: no cytolysin or phage study carries a drinking or craving endpoint	Cells: hepatocytes. Cytolysin kills them, and faecal cytolysin-positive *E. faecalis* tracked AH severity and mortality, and phage targeting cytolytic *E. faecalis* abolished ethanol-induced liver disease in microbiota-humanised mice (M, H)	One side not evaluated — no drinking-side evidence in an alcohol context	The carriage-and-survival association failed independent replication in a prospective cohort (n = 28, underpowered; single country), capping the human side at T4 (H, neg)	NTR-101 oral bacteriophage, ph 1 AH registered but not yet recruiting (NCT06750588, last verified 2025-08)	
IL-22 with REG3G (indole-3-acetic acid, AHR); *IL22*, *REG3G*. Microbial effector (Section 6)	Cells: none shown. Not shown: no IL-22 or REG3G manipulation has a drinking endpoint, and the randomised faecal-transplant signal belongs to the microbiota, not this target	Cells: intestinal type-3 innate lymphoid cells; epithelium (REG3G). Ethanol-associated dysbiosis lowers indole-3-acetic acid, reducing innate-lymphoid-cell IL-22 and intestinal REG3G; restoring either prevents translocation and steatohepatitis, and protection is lost in *Reg3g*-null mice (M)	One side not evaluated — no drinking-side evidence in an alcohol context	Faecal indole-3-acetic acid reduced in patients with AH (H, associative); the agonist's only human trial is open-label with propensity-matched historical controls, so it grades T1-weak and does not raise the tier (H)	F-652 (recombinant IL-22) with acamprosate, ph 2 AH with return to drinking co-primary (NCT07060638)	
GRP with GRPR; *GRP*, *GRPR*. Neuropeptide (Section 5.4)	Cells: not resolved for the alcohol effect; accumbens GRPR+ D2-receptor-	Cells: myeloid cells (GRPR). GRPR is high in alcohol-related	Different experimental systems — and the two sides move in opposite	GRPR raised in alcohol-related steatohepatitis liver and pro-GRP in	None clinical; RH-1402 is a preclinical antagonist	

	expressing projection neurons for a food reinforcer‡. Peripheral bombesin lowers alcohol intake and its antagonist blocks that effect and alone initially raises drinking, with a receptor-family-selective ligand and the site of action unresolved (R); accumbens-specific *Grpr* deletion raises motivation for a food reinforcer (M)‡	steatohepatitis liver with H3K27ac-driven GRP induction, and global and myeloid-specific *Grpr* deletion plus the antagonist RH-1402 reduce injury, steatosis and neutrophil influx (M, HT)	directions	patient mononuclear cells (HT, associative)		

Inline outcome annotations inside an evidence cell. "**neg**" marks a **failed or negative human trial**. "**null**" marks an **animal result without the expected effect** — genetic (knockout or conditional deletion) or pharmacological (an antagonist or agonist that lost or lacked its effect); the null convention outranks the "neg" convention for every animal manipulation. "**uninformative**" marks an interventional study that could not inform, with the reason given in the cell. "**harm**" marks a manipulation that moved the organ the wrong way. "**opposite**" marks a result whose direction contradicts the row's other evidence. These annotations describe a result. **Evidence-source symbols. ‡** the only evidence for this cell comes from a non-alcohol model **or a non-alcohol endpoint** (for example a food reinforcer) (carbon tetrachloride, bile-duct ligation, methionine–choline-deficient diet, high-fat/MASLD, acetaminophen, cholestatic, obesity or cachexia); it may support mechanistic plausibility but is not alcohol evidence. **§** metabolic dysfunction–associated steatohepatitis data, cited as an explicitly labelled liver-side benchmark only, labelled as such. **¶** no human study has yet reported hepatic or nutritional safety or outcome data for this drug class in **alcohol-related cirrhosis**; the retrieved cohorts are in mixed-aetiology or steatohepatitis populations and do not satisfy the population requirement, so the cell is left unfilled rather than inferred. **††** the agent in the phase 2 trial in an alcohol-related liver disease population (NCT06409130, results not published, checked 2026-10-17) is identified as an FGF21 analogue only by **attributed inference**, because the registry record states no molecular target; the only reported fibrosis trial of that agent was in steatohepatitis and was negative (§-labelled). **Species and design badges. M** mouse; **R** rat; **NHP** non-human primate; **H** human study; **HG** human genetics; **HT** human tissue. "ph" = trial phase; "est." = estimated enrolment; "NA" = the registry assigns no drug-development phase; "pp" = percentage points. **Abbreviations (Table S1).** 2-AG, 2-arachidonoylglycerol; AAV, adeno-associated virus; ADRB2, β2-adrenergic receptor; AGER, gene encoding RAGE; AH, alcohol–associated hepatitis; AHR, aryl-hydrocarbon receptor; ALD, alcohol–associated liver disease; ALT, alanine aminotransferase; AMY1R, amylin receptor 1; ASC, apoptosis-associated speck-like protein containing a CARD; AST, aspartate aminotransferase; AUD, alcohol use disorder; CB1, cannabinoid receptor 1; CD8, cluster of differentiation 8; ChREBP, carbohydrate-response-element-binding protein; CNS, central nervous system; CREB, cAMP-response-element-binding protein; CTR, calcitonin receptor; CXCL1, C-X-C motif chemokine ligand 1; CYP2E1, cytochrome P450 2E1; dLS, dorsal lateral septum; EAAT2, excitatory amino-acid transporter 2; FGF15/FGF19/FGF21, fibroblast growth factor 15, 19, 21; FXR, farnesoid X receptor; GDF15, growth differentiation factor 15; GFRAL, GDNF-family receptor α-like; GGT, γ-glutamyl transferase; GIP, glucose-dependent insulinotropic polypeptide; GIPR, GIP receptor; GLP-1R, glucagon-like peptide-1 receptor; GPBAR1, gene encoding TGR5; GRP, gastrin-releasing peptide; GRPR, GRP receptor; G-CSF, granulocyte colony-stimulating factor; H3K27ac, histone H3 lysine-27 acetylation; HMGB1, high-mobility group box 1; IL, interleukin; IL-1R1, interleukin-1 receptor type 1; IRF1, interferon regulatory factor 1; KLB, β-Klotho; LSEC, liver sinusoidal endothelial cell; Ly6C, lymphocyte antigen 6 complex locus C; MASH, metabolic dysfunction–associated steatohepatitis; MASLD, metabolic dysfunction–associated steatotic liver disease; MetALD, metabolic dysfunction–associated alcohol-related liver disease; mGluR5, metabotropic glutamate receptor 5; MR, Mendelian randomisation; NK, natural killer; NLRP3, NLR family pyrin domain containing 3; NOX2, NADPH oxidase 2; NR1H4, gene encoding FXR; NR4A1, nuclear receptor subfamily 4 group A member 1; NTS, nucleus tractus solitarius; OXTR, oxytocin receptor; PDE4, phosphodiesterase 4; PEth, phosphatidylethanol; PKC, protein kinase C; RAGE, receptor for advanced glycation end-products; RAMP1, receptor activity-modifying protein 1; REG3G, regenerating islet-derived protein 3γ; SIRT1, sirtuin 1; SNARE, soluble *N*-ethylmaleimide-sensitive-factor attachment protein receptor; TG, triglyceride; TGR5, Takeda G-protein-coupled receptor 5; TLR4, Toll-like receptor 4; VGLUT3, vesicular glutamate transporter 3; xCT, cystine–glutamate antiporter light chain (SLC7A11); Ca^{2+}, calcium ion. Italicised strings are **gene symbols** and are not separately expanded: human *GLP1R*, *GIPR*, *CALCR*, *FGF21*, *KLB*, *GDF15*, *GFRAL*, *GRM5*, *SLC17A8*, *SLC7A11*, *TLR4*, *HMGB1*, *AGER*, *IL1B*, *NLRP3*, *IL1R1*, *PDE4B*, *CNR1*, *OXTR*, *NR1H4*, *GPBAR1*, *FGF19*, *IL22*, *REG3G*, *GRP*, *GRPR*, *LEAP2*, *GHSR*; mouse *Glp1r*, *Fgf21*, *Gdf15*, *Gfral*, *Grm5*, *Cybb*, *Clec4f*, *Il1r1*, *Nlrp3*, *Grpr*, *Tlr4*, *Nrf2*, *Nox2*, *Cyp7a1*, *Reg3g*, *Gpr65*. Each encodes the protein of the same name as expanded above. *Enterococcus faecalis* and *Lactobacillus reuteri* are species names.

Supplementary Methods S1. Search strings and screening

The seven query families below were run in PubMed through the E-utilities and closed on 17 October 2026, as described in Section 2.2 of the main text. Counts are the primary records each block retrieved after reviews, systematic reviews, meta-analyses, editorials, comments and letters were excluded.

The blocks were, in order: (semaglutide OR liraglutide OR exenatide OR dulaglutide OR tirzepatide OR survodutide OR retatrutide)[tiab], Glp1r[tiab] AND (knockout OR conditional), "hepatic GLP-1 receptor"[tiab] and "lateral septum"[tiab] AND GLP-1, which returned 145 primary records on the drinking side and 166 on the liver side; (pramlintide OR cagrilintide OR salmon calcitonin OR amylin)[tiab], (LEAP2 OR "liver-expressed antimicrobial peptide 2")[tiab], (GOAT OR MBOAT4 OR "ghrelin O-acyltransferase")[tiab], GHSR[tiab] AND (craving OR self-administration), (GDF15 OR MIC-1 OR NAG-1)[tiab] and GFRAL[tiab] AND "area postrema", returning 46 and 11 for the ghrelin terms and 12 and 16 for GDF15; FGF21[tiab] AND (ethanol OR alcohol OR preference OR "sweet taste"), (KLB OR "beta-Klotho" OR "β-Klotho")[tiab], (zalfermin OR efruxifermin OR pegozafermin OR "PF-05231023" OR NNC0194-0499)[tiab], (FXR OR NR1H4 OR obeticholic OR INT-787)[tiab] and (TGR5 OR GPBAR1 OR FGF19)[tiab], returning 67 and 170 for FGF21 and 505 for the bile-acid terms; (mGluR5 OR GRM5 OR mavoglurant OR MPEP OR MTEP OR fenobam OR GET73)[tiab], (VGLUT3 OR SLC17A8 OR "vesicular glutamate transporter")[tiab] and (GRP OR GRPR OR "gastrin-releasing peptide")[tiab], returning 302 and 53; and cytolysin[tiab] AND (Enterococcus OR hepatitis), (bacteriophage OR phage)[tiab], (REG3G OR REG3B OR "regenerating islet-derived")[tiab], "fecal microbiota transplantation"[tiab], (TLR4 OR HMGB1)[tiab], (anakinra OR canakinumab OR "IL-1 receptor antagonist")[tiab], (PDE4B OR apremilast OR ibudilast OR roflumilast)[tiab] and (CB1 OR CNR1 OR cannabidiol OR rimonabant OR "peripherally restricted")[tiab], returning 1,367 and 144 for the microbial terms, 793 and 126 for the innate-immune terms, 57 and 25 for PDE4 and 84 and 21 for CB1.

The axis family ran "gut-liver-brain axis"[tiab], "gut-brain axis"[tiab], "vagal afferent\ *"[tiab], "intestinal permeability"[tiab], hepatokine*\[tiab] and MetALD[tiab] against the drinking block, returning 90; the endpoint family ran phosphatidylethanol[tiab], PEth[tiab] AND (validity OR sensitivity), "enhanced liver fibrosis"[tiab] OR "ELF score"[tiab], "co-primary"[tiab] AND alcohol, "target trial emulation"[tiab] AND alcohol, "chronic-plus-binge"[tiab] OR "NIAAA model"[tiab], and "intermittent access"[tiab] AND ethanol AND (fibrosis OR liver). The window was 1 January 2021 to 17 October 2026 by publication date, with review, systematic-review, meta-analysis, editorial, comment and letter record types excluded from the counts above; earlier work was retrieved only where it is the origin of a concept the review needs, as with the bombesin pharmacology of Section 5.4 and the forced-feeding and free-choice models of Section 7.

Candidates retrieved but not entered in the matrix. Three are worth naming, because each fails the dual-target condition in a different way, and in each case the searches did return something. **PCSK9** has primary hepatic evidence in an alcohol model, but the only retrieved source placing it on the drinking side is a review proposing the axis rather than primary data, so it has one side and not two (retrieved records PMID 33919550, 31748600, 35162992). **Lipocalin-2** is the mirror image: primary liver-side evidence in alcohol-related injury, with no brain-side evidence retrieved (PMID 26682726, 32968110). **GOAT** is the enzyme that acylates ghrelin rather than a node of its own; it is appraised inside the ghrelin target in Section 3.4, where acylation is discussed, so listing it separately would count one system twice. PMIDs here identify records the search returned and are not reference-list entries.
